# Interfering with retrotransposition by two types of CRISPR effectors: Cas12a and Cas13a

**DOI:** 10.1038/s41421-020-0164-0

**Published:** 2020-05-19

**Authors:** Niubing Zhang, Xinyun Jing, Yuanhua Liu, Minjie Chen, Xianfeng Zhu, Jing Jiang, Hongbing Wang, Xuan Li, Pei Hao

**Affiliations:** 1 Key Laboratory of Synthetic Biology, CAS Center for Excellence in Molecular Plant Sciences, Institute of Plant Physiology and Ecology, Chinese Academy of Sciences, Shanghai, 200032 China; 2 School of Life Sciences, Henan University, 475000 Kaifeng, Henan China; 3 Key Laboratory of Molecular Virology and Immunology, Institute Pasteur of Shanghai, Chinese Academy of Sciences, Shanghai, 200031 China; 4 Department of Physiology, Michigan State University, East Lansing, MI USA

**Keywords:** Molecular biology, Cell biology

## Abstract

CRISPRs are a promising tool being explored in combating exogenous retroviral pathogens and in disabling endogenous retroviruses for organ transplantation. The Cas12a and Cas13a systems offer novel mechanisms of CRISPR actions that have not been evaluated for retrovirus interference. Particularly, a latest study revealed that the activated Cas13a provided bacterial hosts with a “passive protection” mechanism to defend against DNA phage infection by inducing cell growth arrest in infected cells, which is especially significant as it endows Cas13a, a RNA-targeting CRISPR effector, with mount defense against both RNA and DNA invaders. Here, by refitting long terminal repeat retrotransposon Tf1 as a model system, which shares common features with retrovirus regarding their replication mechanism and life cycle, we repurposed CRISPR-Cas12a and -Cas13a to interfere with Tf1 retrotransposition, and evaluated their different mechanisms of action. Cas12a exhibited strong inhibition on retrotransposition, allowing marginal Tf1 transposition that was likely the result of a lasting pool of Tf1 RNA/cDNA intermediates protected within virus-like particles. The residual activities, however, were completely eliminated with new constructs for persistent crRNA targeting. On the other hand, targeting Cas13a to Tf1 RNA intermediates significantly inhibited Tf1 retrotransposition. However, unlike in bacterial hosts, the sustained activation of Cas13a by Tf1 transcripts did not cause cell growth arrest in *S. pombe*, indicating that virus-activated Cas13a likely acted differently in eukaryotic cells. The study gained insight into the actions of novel CRISPR mechanisms in combating retroviral pathogens, and established system parameters for developing new strategies in treatment of retrovirus-related diseases.

## Introduction

The CRISPR (clustered regularly interspaced short palindromic repeat) and CRISPR-associated systems (Cas) are adaptive immunity mechanism against invading viruses or plasmids, which originated and evolved in bacteria and archaea^1,2^. In recent years, they have emerged as an important and promising tool being explored in combating foreign retroviral pathogens and in inactivating endogenous retroviruses (ERVs) for organ transplantation. CRISPR was considered advantageous to the previous technologies, such as TALEN^3^, ZFN^4,5^, and RNAi^6,7^, as it is easier to handle, more efficient, and less expensive. Both endogenous and exogenous retroviruses are known to be causes of major health issues for people, and risk factors for organ transplantation and xenotransplantation^8–11^. Well-known examples include acquired immune deficiency syndrome (AIDS) and HIV-associated neurological disorders (HAND) caused by HIV-1^12–15^, leukemia/lymphoma and autoimmune diseases induced by Human T-lymphotropic virus type-1 (HTLV-1)^16^, and cancer and autoimmunity associated with the less known Human Endogenous Retrovirus K (HERV-K)^17,18^. Among CRISPR systems that have been tested, class 2 type II CRISPR-Cas9 was the first engineered to combat retroviral pathogens in animals and plants. CRISPR-Cas9 was reprogrammed to targeting HIV-1 under a Tat-feedback regulation, leading to ablation of HIV-1 at a very early stage of replication in the course of the acute infection, and in latently infected cells^19^. CRISPR-Cas9 was found to be potent for inactivating latent HIV-1 in infected cells by targeting HIV-1 essential genes or long terminal repeat (LTR)^20,21^. Cas9 was used as an efficient research tool to perturb retroviral LTRs in human cells to determine their long-range effects on gene regulation^22^. CRISPR-Cas9 was also explored for disabling of ERVs in pigs to create ERV-inactivated animals for xenotransplantation, which is an essential step to address clinical safety issue^23^. The targeted deletion of retrotransposon *Tos17* in rice using CRISPR-Cas9, resulted in the new generation of plant free of active *Tos17* retrotransposon^24^.

The recently emerged class 2 type V CRISPR-Cas12a (also called Cpf1) has some intrinsic features that make it an important alternative to Cas9^25–29^. Cas12a has a different capacity for targeting AT-rich genomic regions, which expands that of Cas9, due to its different restrictive requirement for protospacer adjacent motif (PAM) sequence^25,30^. It possesses endogenous RNase activity enabling the generation of single or multiplex CRISPR RNA (crRNA) without the need for involving other RNA nucleases for processing^31–34^. Besides, Cas12a requires a much shorter crRNA (~41 nucleotides)^25,35^ than the fused crRNA-tracrRNA of Cas9 (~100 nucleotides)^36,37^, thus simplifying its use and assay development, such as chemical synthesis and modification of crRNA^38^. Further, Cas12a was reported to have better targeting precision than Cas9 in a number of genome editing experiments^26,39^. Despite all these superior attributes, there has been no reported study of Cas12a application in retrovirus/retrotransposon intervention. Therefore, we are severely limited in knowledge and capacity in applying this valuable CRISPR tool for combating retrovirus.

More recently, the class 2 type VI CRISPR-Cas13 group of effectors were discovered and found to possess the unique ability to specifically target and cleave single-stranded RNA rather than double-stranded DNA substrates^40–43^. They present the novel capacity to bind RNA molecules guided by crRNAs that contain a fixed direct repeat (DR) and variable spacer sequences. In addition, some members of them, e.g., LshCas13a^40^, were proved to be able to process pre-crRNA into crRNAs, thus facilitating their application by simplifying the production of crRNAs. Since its first discovery in 2016, CRISPR-Cas13 has seen actions in nucleic acid detection^44^, gene transcript tagging and tracking^45^, and gene transcript editing and mutation correction^46,47^, etc. Many efforts have also been focused on utilizing Cas13a in engineering RNA virus resistance in plants. LshCas13a was shown to confer resistance to TuMV infections in *Nicotiana benthamiana* and *Arabidopsis thaliana* in transient assays and in stable-expression lines^48,49^. Immunity was also established using Cas13a in potato against the RNA virus, Potato virus Y (PVY), and in rice against both Southern Rice Black-Streaked Dwarf Virus (SRBSDV) and Rice Stripe Mosaic Virus (RSMV)^50,51^.

Lately CRISPR-Cas13a was reported to provide hosts with immunity against DNA phage infection by halting the growth of infected cells in *Listeria* populations (termed passive protection), in addition to its ability to target and cleave RNA virus^52^. The nonspecific RNase activity of activated Cas13a was believed to be involved in the passive protection mechanism, which aborted the infectious cycle in bacterial populations and provided cross-protection for host cells against mutant escaper virus as well. This passive protection mechanism is particularly significant and powerful in that it endows Cas13, an RNA-targeting CRISPR effector, with mount defense against both RNA and DNA invaders. To build on this, it is reasonable to suspect that CRISPR-Cas13a may provide a potent defense against retroviruses, which transit between DNA and RNA forms in their life cycle, by either directly targeting RNA intermediates, or halting viral propagation with the passive protection mechanism. A particular aim of the current study was to test this hypothesis and investigate the system parameters in applying Cas13a to retrovirus interference, which has not been reported before.

LTR retrotransposons and retroviruses share many common features regarding their genome structure, replication mechanism, and life cycle^53,54^. Both LTR retrotransposons and retroviruses replicate via reverse transcription and propagate by integrating into the host’s genome, which rely on the host transcription and translation machinery. Most LTR retrotransposons lose extracellular mobility and lack a functional envelope gene (ENV), but contain gag, protease (PR), reverse transcriptase (RT), and integrase genes (IN) that are arranged in a polycistron open reading frame (ORF), flanked by LTRs. LTR retrotransposons are abundant in genomes of some animals and plants. For example, LTR retrotransposons occupy ~8% and ~10% of the human and mouse genomes, respectively^55,56^. In the current study, using LTR retrotransposon Tf1 as a model system^57,58^, we explored the two CRISPR systems: class 2 type V CRISPR-Cas12a and type VI CRISPR-Cas13a, in interference with Tf1 retrotransposition using different mechanisms of CRISPR action. To test CRISPR-Cas12a, we first overcame the lack of Cas12a system in *Schizosaccharomyces pombe* by implementing and optimizing Cas12a gene editing systems in *S. pombe*. Second, we constructed a series of Tf1-splicing reporters by refitting the Tf1 retrotransposon with artificial introns. This system enabled the Tf1 cDNA intermediates that were reverse-transcribed from spliced-RNA, to be specifically targeted by Cas12a with designed crRNAs. While both Cas12a and Cas13a were robust in intervention with Tf1 retrotransposition, they had varying efficiencies and unequal characters in inhibition of Tf1 transposition, owing to the intrinsic differences in their nature of molecular action. By implementing and testing the versatile systems of CRISPR-Cas12a and -Cas13a, we established their system parameters for the different action mechanisms on Tf1 interference in *S. pombe*.

## Results and discussion

### Implementing and optimizing CRISPR-Cas12a editing system in *S. pombe*

**Fig. 1:**
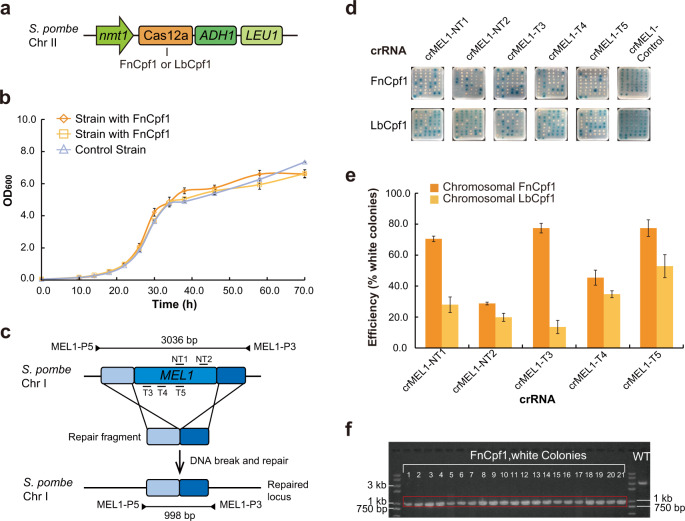
Implementation of Cas12a editing system in *S. pombe* and editing of *MEL1* gene. **a** Schematic representation of the *S. pombe* chromosomal locus with integrated Cas12a constructs. **b** Growth in liquid culture media (EMM + uracil) of *S. pombe* strains expressing FnCpf1 or LbCpf1, compared to the control strain with blank insertion at the same locus. They had an average double time of 4.1, 4.6 and 4.5 h, respectively. Data are means ± SEM (*n* = 4 independent experiments). **c** Schematic representation of *MEL1* gene editing. crRNAs were designed to target five sites (NT1, NT2, T3, T4, and T5) within *MEL1* gene. The DNA cut by Cas12a is repaired with the repair fragment by HR. The arrows denote the primers, MEL1-P5 and MEL1-P3, and their PCR products for diagnostic experiments. **d** The editing efficiency on *MEL1* gene with five different crRNAs, estimated by formation of white- and blue-colored colonies, assayed on agar plates containing the X-α-Gal. **e** Efficiency of genome editing on *MEL1* gene with five different crRNAs. Values are mean ± SEM, *n* = 3. **f** Diagnostic PCR products of *mel1* mutants (white colonies) generated from FnCpf1 editing, showing 998-bp in size. They are compared with the wild-type (WT) product, 3036-bp in size. Primer sets MEL1-P5 and MEL1-P3 (**c**) were used for PCR.

To implement Cas12a system in *S. pombe* and optimize its gene editing efficiency, expression constructs of *Francisella novicida* Cpf1 (FnCpf1) and *Lachnospiraceae bacterium* ND2006 Cpf1 (LbCpf1) were generated with pDUAL-HFF1 vector (Supplementary Fig. S1). The expression constructs of FnCpf1 and LbCpf1 were placed in the *LEU1* locus in *S. pombe* chromosome II by homologous recombination (HR)^59^ (Fig. 1a and Supplementary Fig. S2). The impact of FnCpf1 or LbCpf1 on growth of *S. pombe* was assessed. The transformed strains were found to grow at rates similar to the isogenic control strain in Edinburgh minimal medium (EMM) (Fig. 1b). Therefore, the presence of a single copy of FnCpf1 or LbCpf1 gene had no detectable impact on the growth of *S. pombe* cells.

The genome editing efficiency of FnCpf1 and LbCpf1 was determined by editing *MEL1* (SPAC869.07c) gene through introducing *MEL1*-targeting crRNA constructs (Supplementary Fig. S3a) into FnCpf1- or LbCpf1-expressing strains. The *MEL1* gene encodes the α-galactosidase in *S. pombe*, which is secreted and readily assayed on agar plates containing the colorimetric substrate 5-bromo-4-chloro-3-indolyl-α-galactopyranoside (X-α-Gal)^60,61^. While wild-type *S. pombe* colonies displayed blue color, the loss-of-function *mel1* mutants resulted in white colonies on X-α-Gal plates. We designed two nontargeting control crRNAs, and ten crRNAs (five for each Cas12a) for targeting FnCpf1 and LbCpf1 to five different regions of *MEL1* gene in *S. pombe*, three on the template strand (T strand) and two on the nontemplate strand (NT strand) in its ORF (Fig. 1c and Supplementary Fig. S3b). The editing efficiency for FnCpf1 and LbCpf1 was estimated by comparing the numbers of white-colored and blue-colored colonies. The two Cas12a had varying editing efficiencies on *MEL1*, ranging from 29 to 77% for FnCpf1, and 14 to 55% for LbCpf1, respectively (Fig. 1d, e and Supplementary Fig. S3b), compared to 50−98% editing efficiency for Cas9 in targeting a different gene, *ADE6* in *S. pombe*^62^. While both Cas12a worked effectively in *S. pombe*, FnCpf1 apparently generated *mel1* mutants at a higher rate than LbCpf1 for all the five targeted sites. Thus, for the current study, we chose FnCpf1 for subsequent experiments in *S. pombe*. It was known that the editing efficiency of Cas12a could be influenced by many factors in different organisms, e.g., base contents, sequence bias, PAM sequences, epigenetic state of targets, etc^31^. For example, it was previously found in rice that LbCpf1 exhibited a better editing efficiency than FnCpf1 on three endogenous genes^34^.

The loss-of-function *mel1* mutants generated from Cas12a editing were further examined by PCR analysis and sequencing of *mel1* PCR products (Fig. 1f and Supplementary Fig. S4). The PCR products of 41 randomly picked *mel1* mutants from either FnCpf1 or LbCpf1 editing indicated that *mel1* mutants were all repaired via HR. While repairing via both HR and nonhomologous end joining (NHEJ) for CRISPR-Cas9 editing in *S. pombe* were documented previously^62^, in the case of Cas12a editing, however, NHEJ was not observed to be involved in *MEL1* editing by either FnCpf1 or LbCpf1 in *S. pombe*. The observed high efficiency for HR may be the result of long homologous arms (~500 bp) as it was indicated that increase in HR efficiency was associated with longer homologous arms^63–65^.

To optimize the Cas12a system for editing in *S. pombe*, we further varied FnCpf1 and LbCpf1 expression levels using episomal multicopy plasmid^66^ (Supplementary Fig. S5) and examined their efficiencies targeting the same five sites on *MEL1* gene. The increased LbCpf1 expression (while crRNA levels remained constant) in *S. pombe* resulted in an increased editing efficiency for four of the five targeted sites in *MEL1* gene of crRNA (Supplementary Fig. S6c). This observation in *S. pombe* is similar to that of Cas9 in previous studies, where Cas9 expression levels were found to be positively correlated with editing efficiency^67,68^. To our surprise, the increased FnCpf1 expression in *S. pombe* resulted in a decreased editing efficiency for four of the five targeted sites in *MEL1* gene of crRNA (Supplementary Fig. S6b). To rule out the possibility that the reduced editing efficiency was caused by possible toxicity of high FnCpf1 level in *S. pombe* cells, we further examined the growth of *S. pombe* strains with either FnCpf1-expressing multicopy plasmid (pDUAL-HFF1-FnCpf1) or the control plasmid (pDUAL-HFF1). They were found to grow at similar rates in EMM with glucose as carbon source (Supplementary Fig. S7), indicating no toxicity on *S. pombe* growth with increased FnCpf1 expression.

### Construction of multiple Tf1-splicing reporters for retrotransposition interference analysis in *S. pombe*

**Fig. 2:**
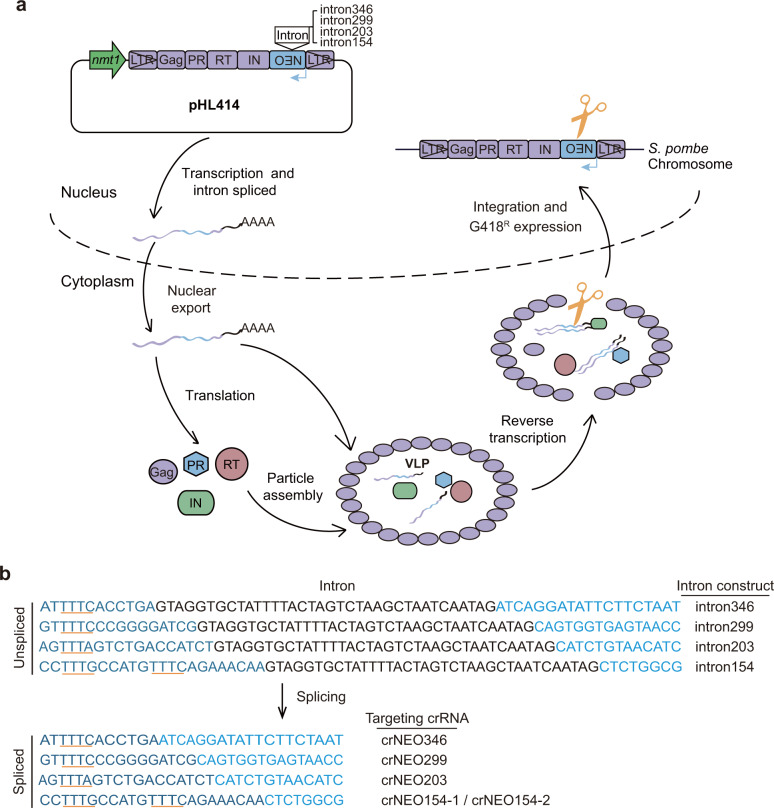
Design and construction of Tf1-splicing reporter system for retrotransposition. **a** Schematic representation of life cycle for Tf1-splicing reporter. The *NEO* gene was placed in Tf1 genome upstream of 3′-LTR, but would transcribe in the opposite orientation to Tf1 transcription (denoted by a blue arrow). Four different artificial introns were designed to be inserted in *NEO* gene, respectively, aligned in the same orientation to that of Tf1. Scissors denotes Cas12a-targeting sequences of *NEO* gene formed from splicing. *nmt1* *nmt1* promoter, Gag gag protein, PR protease, RT reverse transcriptase, IN integrase, *NEO* G418 resistance gene. **b** Schematic representation of four *NEO* gene constructs with artificial intron (black letters) in different insertion sites and their splicing products. The sequences formed via splicing are targeted by designed crRNAs (denoted to the right). The five PAM regions for Cas12a targeting are underlined in orange.

The retrotransposon Tf1 can mobilize in *S. pombe* in a manner similar to retroviruses, through synthesis of RNA intermediates, virus-like particle (VLP) formation and reverse transcription, followed by integration of DNA intermediates into host genome^54^. To facilitate analysis of interference with retrotransposition by CRISPR-Cas12a, we constructed Tf1-G418^R^ retrotransposition reporter systems in *S. pombe*, which were modified based on scheme from previous studies^47,69^. A geneticin (G418) resistance gene *NEO* had been placed in Tf1 genome just upstream of 3′-LTR, but in the opposite orientation to Tf1 transcription. Then, by design, we inserted an artificial intron element into the ORF of *NEO* gene in opposite orientation to the *NEO* transcript, which would align in the same orientation to that of Tf1 (Fig. 2a). When the artificial intron is removed upon splicing of Tf1 transcripts, the resulting retrotransposed Tf1 would make host cells G418-resistant. The reporter system was designed to allow the original Tf1 (carried by plasmids) and reverse-transcribed Tf1 DNA (before or after integration into *S. pombe* genome) to be distinguished and specifically targeted. For Cas12a-targeting reverse-transcribed Tf1 cDNA, we chose four sites with nearby PAM sequences in the *NEO* gene for inserting the artificial intron, respectively (Fig. 2b; Materials and methods), from which splicing products could be specifically targeted with designed crRNAs.

### Interference with Tf1 retrotransposition by CRISPR-Cas12a resulted in marginal transposition activities

**Fig. 3:**
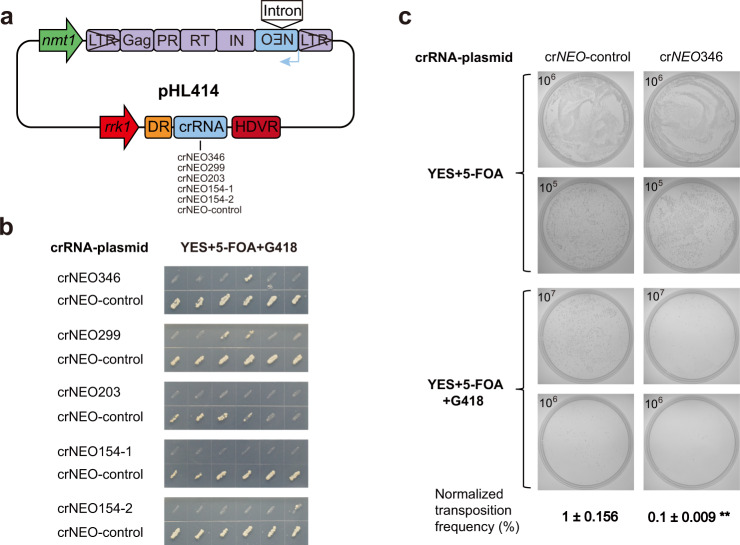
Interference of Tf1 retrotransposition by CRISPR-Cas12a. **a** Schematic representation of six crRNA constructs for Cas12a targeting, for which the crRNA expression cassette was inserted into the Tf1-carrying pHL414 plasmid (Supplementary Table S4). crNEO-control contains nontargeting random sequence. *rrk1* *rrk1* promoter, DR direct repeat of crRNA, HDVR hepatitis delta virus ribozyme. **b** “Transfer-and-patch” assay for detection of Tf1 retrotransposition using plates containing YES media with 5-FOA and G418. The effects of the five different crRNAs and their control crNEO-control were shown side by side. **c** Estimation of Tf1 retrotransposition efficiency by colony-forming assay. Cells were plated in replicates to YES plates with either 5-FOA only or 5-FOA + G418. The values in the upper-left corner indicate the diluted cell concentration (cells/mL), from which 100 μL were plated (Materials and methods). Transposition efficiencies for crRNA crNEO346 and crNEO-control were estimated by comparing the number of formed colonies between the two plate conditions (Supplementary Data S2). The efficiency values were then normalized with that of crNEO-control as “1%”. Data are means ± SEM (*n* = 3 independent experiments; **P < 0.01 by Student’s *t* test against crNEO-control).

To investigate interference with Tf1 retrotransposition by CRISPR-Cas12a, we designed five crRNA constructs that specifically targeted the four sequence fragments (generated via splicing) of DNA intermediates, respectively (Fig. 2b). CRISPR-Cas12a was previously shown to act on DNA substrate, and had no editing activity on RNA molecules^70^. The five crRNAs for FnCpf1 were placed in the same reporter plasmid under the control of *rrk1* promoter, respectively^62^ (Fig. 3a). We then transformed these reporter plasmids into the FnCpf1-carrying *S. pombe* strain that we generated earlier.

Tf1 transposition was initiated by withdrawing thiamine from media, prompting Tf1 transcription under *nmt1* promoter. Retrotransposition efficiency was assayed by counting *S. pombe* colonies in YES plates containing 5-fluoroorotic acid (5-FOA) and G418^69^. Using the “transfer-and-patch” assay^69^ and comparing to the nontargeting control crRNA (crNEO-control), we observed dramatically reduced Tf1 retrotransposition with all five targeting crRNAs (Fig. 3b), indicating that Cas12a (FnCpf1) strongly inhibited Tf1 retrotransposition. While there was almost no Tf1 transposition with crRNAs crNEO203, crNEO154-1 and crNEO154-2, residual levels of retrotransposition occurred with crNEO299 and crNEO346.

To more accurately estimate the inhibition on Tf1 retrotransposition, we then measured the retrotransposition frequency for crRNA crNEO346 that had the least inhibitory effect among the five, using the colony-forming assay^69^. A normalized transposition frequency of 0.1 ± 0.009% was recorded for crNEO346. Compared to that of 1 ± 0.156% for the nontargeting control crRNA, the result represented an inhibition of 90% on Tf1 retrotransposition (Fig. 3c).

### Prolonged crRNA targeting eliminated residual Tf1 retrotransposition by CRISPR-Cas12a

**Fig. 4:**
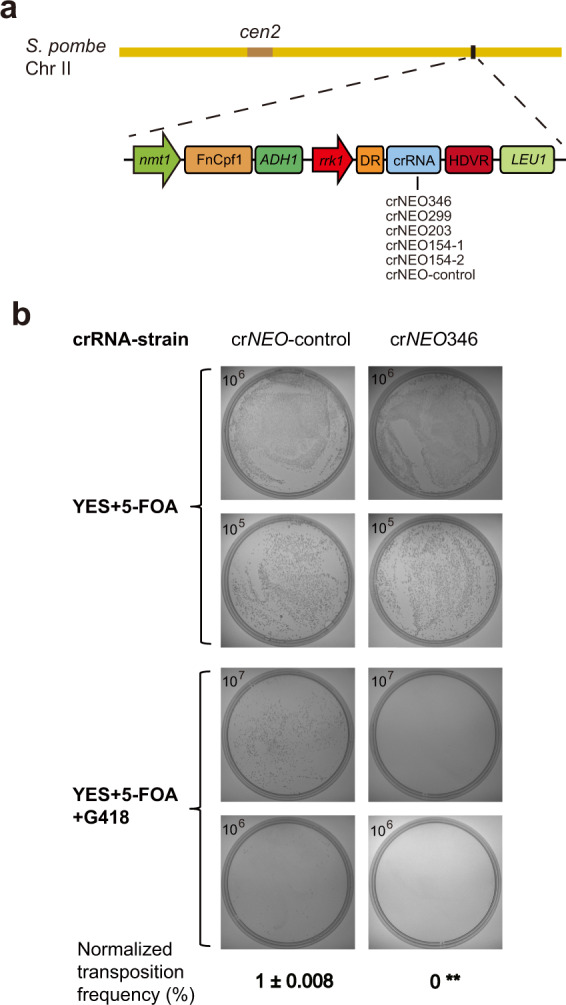
Prolonged crRNA targeting eliminated residual Tf1 retrotransposition by Cas12a. **a** Schematic representation of six different crRNA constructs integrated into chromosome II in the six new *S. pombe* strains. crNEO-control contains nontargeting random sequence. *cen2* centromere. **b** Estimation of Tf1 retrotransposition efficiency by colony-forming assay. Cells were plated in replicates to YES plates with either 5-FOA only or 5-FOA + G418. The values in the upper-left corner indicate the diluted cell concentration (cells/mL), from which 100 μL were plated (Materials and methods). Transposition efficiencies for the persistent presence of crNEO346 and crNEO-control were estimated by comparing the number of formed colonies between the two plate conditions (Supplementary Data S2). The efficiency values were then normalized with that of crNEO-control as “1%”. Data are means ± SEM (*n* = 3 independent experiments; **P < 0.01 by Student’s *t* test against crNEO-control).

CRISPR-Cas12a was shown to strongly inhibit Tf1 retrotransposition, allowing residual levels of transposition activities for some crRNA constructs. We reasoned that the “leaked” residual activities could be due to a lasting pool of Tf1 RNA/DNA intermediates, more persistent than crRNA and likely protected within VLPs, that remain to be further explored. That is, after the *S. pombe* cells were transferred to 5-FOA plates to remove pHL414 plasmids carrying Tf1, the crRNAs transcribed from the same vectors also wound down in the cells. As a result, the remnant Tf1 intermediates could enable residual retrotransposition at the time when crRNA molecules were depleted. We sought to test whether we can remove the residual Tf1 retrotransposition with prolonged crRNA targeting. Therefore, we generated six new *S. pombe* strains by integrating the five targeting and one nontargeting control crRNA cassettes into chromosome II at the site near FnCpf1 gene, respectively (Fig. 4a). These constructs enabled persistent crRNA expression even after Tf1-carrying plasmids were lost by 5-FOA treatment. The results showed that the persistent presence of the five crRNAs led to complete elimination of the residual Tf1 transposition activities by Cas12a (Supplementary Fig. S8). We also quantitatively assessed the Tf1 retrotransposition frequencies for crRNA crNEO346. Its normalized transposition frequency was reduced to 0 (100% inhibition), in comparison to the nontargeting crRNA control that stood at 1 ± 0.008% (Fig. 4b).

### Interfering with Tf1 retrotransposition by CRISPR-Cas13a via targeting its RNA intermediates

**Fig. 5:**
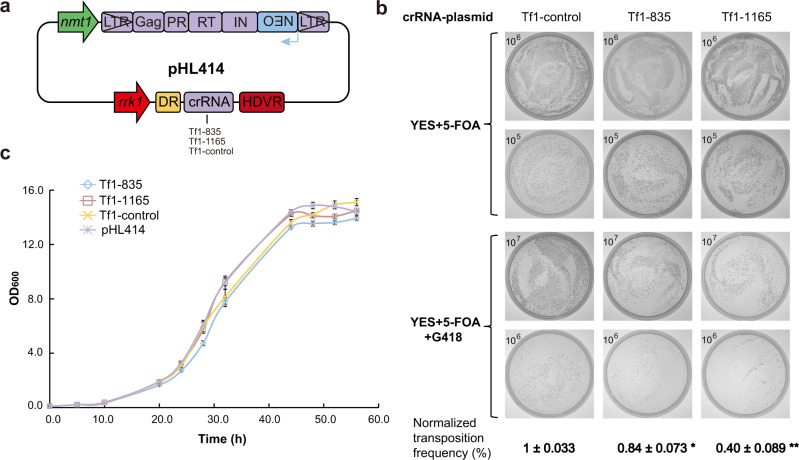
Interfering with Tf1 retrotransposition by CRISPR-Cas13a via targeting its RNA intermediates. **a** Schematic representation of three crRNA constructs for Cas13a targeting, for which the crRNA expression cassette was inserted into the Tf1-carrying pHL414 plasmid (as in Fig. 3a) (Supplementary Table S4). Tf1-control contains nontargeting random sequence. **b** Estimation of Tf1 retrotransposition efficiency by colony-forming assay. Cells were plated in replicates to YES plates with either 5-FOA only or 5-FOA + G418. The values in the upper-left corner indicate the diluted cell concentration (cells/mL), from which 100 μL were plated (Materials and methods). Transposition efficiencies for crRNAs Tf1-835, Tf1-1165 and Tf1-control were estimated by comparing the number of formed colonies between the two plate conditions (Supplementary Data S2). The efficiency values were then normalized with that of Tf1-control as “1%”. Data are means ± SEM (*n* = 3 independent experiments; *P < 0.05, **P < 0.01 by Student’s *t* test against Tf1-control). **c** Growth in liquid culture media (EMM) of LshCas13a strains with either pHL414 plasmid containing Tf1 reporter or pHL414 plasmids containing Tf1 reporter and different crRNA constructs. The strains in the assay were not treated with 5-FOA, for which Cas13a was continually activated in the presence of Tf1 transcripts and targeting crRNAs. The strains containing plasmids Tf1-835, Tf1-1165, crRNA-control or the pHL414 plasmid had an average double time of 5.1, 4.8, 4.8 and 4.7 h, respectively. Data are means ± SEM (*n* = 4 independent experiments).

Tf1 undergoes retrotransposition in *S. pombe* via RNA intermediates^57,58,71^, which are important target for intervention. LshCas13a was shown to have editing activity on single-strand RNA but not on DNA molecules^45^. We hence investigated CRISPR-Cas13a for interference with Tf1 retrotransposition. We took use of the LshCas13a-carrying *S. pombe* strain that we generated previously^47^, for which a single copy of LshCas13a gene was integrated in chromosome II at the *LEU1* locus. The Tf1 retrotransposition reporter plasmid (pHL414) was modified to contain crRNAs for LshCas13a targeting. Three crRNA constructs, two targeting different regions of Tf1 transcripts and one nontargeting control, were placed under the control of *rrk1* promoter, respectively (Fig. 5a). The modified plasmids were then transformed into the LshCas13a-carrying *S. pombe* strain.

After withdrawing thiamine from culture media to initiate Tf1 retrotransposition, the transposition frequencies for different crRNAs were estimated using the colony-forming assay in YES plates containing 5-FOA with or without G418. Compared to the normalized transposition frequency of 1.0 ± 0.033% for the control strain having the nontargeting control crRNA, the strains containing targeting crRNAs Tf1-835 or Tf1-1165, displayed reduced normalized transposition frequency of 0.84 ± 0.073% or 0.40 ± 0.089%, respectively (Fig. 5b). These values represented 16% and 60% inhibition of Tf1 retrotransposition activity, respectively, compared to that of the control strain. The varying effect of crRNAs Tf1-835 and Tf1-1165 was likely due to their different capacities for targeting Cas13a toward Tf1 RNA intermediates, as it had been shown previously that targeting efficiency for Cas13a-crRNA varied largely owing to many factors, especially the secondary structure of RNA targets that influenced crRNA binding^40,47^. Therefore, identifying and employing high-capacity crRNAs became critically important when applying CRISPR-Cas13 techniques to retrovirus intervention. Our results indicated that acting at RNA intermediates within the life cycle of retrovirus is a promising option for intervention by CRISPR-Cas13a.

### Activation of CRISPR-Cas13a by Tf1 transcripts did not induce cell growth arrest in *S. pombe*

CRISPR-Cas13a was recently reported to offer immunity to bacterial hosts against phage infection by inducing growth arrest of infected hosts (termed passive protection), in addition to directly targeting invading virus^40,52^. The mechanism was related to the nonspecific RNase activity of activated Cas13a, which aborted the infectious cycle by stopping phage proliferation, and also to provide cross-protection for hosts against mutant escaper viruses. However, it remained an open question whether the same protection mechanism for Cas13a was involved in eukaryotic hosts.

We have shown that, in the presence of crRNAs Tf1-835 or Tf1-1165, Cas13a significantly inhibited Tf1 from retrotransposition in *S. pombe*. It was imperative for us to test whether cell growth arrest (or growth slowdown) took place with Cas13a activation under these conditions. To look for hint of growth arrest of *S. pombe* cells, we resorted to measuring their growth rates in liquid culture media. However, differently from that for measuring Tf1 retrotransposition frequency (Fig. 5b), in this assay after initiating Tf1 retrotransposition and crRNA production by withdrawing thiamine, no 5-FOA was used to remove Tf1-carrying plasmids from the cells. Thus, throughout culture and measurement, Cas13a was continuously activated in the presence of Tf1 transcripts and targeting crRNAs (Fig. 5c). As a control in the assay, constructs with no crRNA (pHL414) or a nontargeting crRNA (Tf1-control) were used to compare their growth rates. The *S. pombe* strains containing Tf1-targeting crRNAs, Tf1-835 or Tf1-1165, were observed to have similar growth rate with those containing pHL414 or the nontargeting crRNA (Tf1-control) (Fig. 5c). Therefore, no evidence was found for induced cell growth arrest in *S. pombe* cells with continued activation of Cas13a by Tf1 transcripts. We further examined the ending *S. pombe* populations from these liquid cultures for the presence of selection markers for Cas13a gene and Tf1/crRNA-carrying plasmid, which was validated by colony formation on selective media plates (Supplementary Fig. S9). These results indicated that, differently from that in bacterial hosts, where Cas13a activation also provided passive protection by halting the growth of infected cells, the sustained Cas13a activation by Tf1 in *S. pombe* did not cause cell growth arrest in the eukaryotic host. It is likely that virus-activated Cas13a acted differently in the eukaryotic cells than in bacterial hosts. This agrees with the previous observation that the nonspecific RNase activity of activated Cas13a found in bacteria was otherwise undetected in mammalian cells^45^. By establishing the two CRISPR systems taking advantage of two different mechanisms to intervene with retrotransposition in eukaryotic cells, we gained novel insight into the molecular actions of these CRISPR tools in combating retroviral pathogens, and explored potentially new strategies and system parameters for developing therapeutic applications in treatment of retrovirus-related diseases.

## Materials and methods

### Materials and general molecular biology techniques

All oligonucleotides (Supplementary Data S1) were synthesized by Genwiz Biotech (Suzhou, China). Plasmids, *E. coli* strains, and *S. pombe* strains in this study are listed in Supplementary Tables S1−5. Sanger sequencing was performed by TsingKe Biotech (Shanghai, China).

PCR was performed using KOD-FX DNA polymerase (TOYOBO) or Hieff^TM^ PCR Master MixTaq (Yeasen). DNA fragments were separated on agarose gels and were extracted using the Gel Extraction Kit (OMEGA). Plasmid DNA was extracted using the Plasmid Mini Kit I (OMEGA). Cloning construction was performed using restriction endonucleases and T4 DNA Ligase (New England Biolabs), or the ClonExpress® II One Step Cloning Kit (Vazyme). Reverse transcription was performed on total RNA using the RevertAid First Strand cDNA Synthesis Kit (Thermo Scientific) and oligo (dT) primers.

The competent cells of *E.coli* DH5α (Takara Biotechnology Co., Ltd.) were used for chemical transformations for the purpose of plasmid construction and molecular cloning. G418 (geneticin), thiamine (Vitamin B1) and 5-FOA were purchased from Abcam and TCI Development Co., Ltd. All other chemicals were from Sigma-Aldrich (Shanghai, China) if not indicated otherwise.

### *S. pombe* strains, culture and transformation

The *S. pombe* strain FY7652 (*h-leu1-32 ura4-D18*) (National BioResource Project, Osaka, Japan) was used to generate derived strains by transformation with various constructed plasmids or linear DNA (Supplementary Tables S1 and S3−S5). Yeast strains were grown in YES^72^ media (5 g/L yeast extract and 30 g/L glucose supplemented with 50 μg/mL uracil and/or 50 μg/mL leucine) at 32 °C either on plates or in liquid medium with 220 rpm agitation.

Yeast transformation was generally carried out with 500 ng of linearized DNA or 100 ng of circular plasmid. *S. pombe* cultures were first grown to mid-log phase, before cells were collected and transformed using the Lithium Acetate/PEG/Heat shock protocol^72^. Transformants were selected on EMM^72^ supplemented with 50 μg*/*mL uracil or 50 μg/mL leucine. Transformed colonies usually appeared on medium plates after incubation at 32 °C for 2−4 days. The pombe glutamate medium (PMG)^72^ (3 g/L potassium hydrogen phthalate, 2.2 g/L Na_2_HPO_4_, 3.75 g/L l-glutamic acid, monosodium salt, 20 g/L glucose, 20 mL/L of 50× salt stock, 1 mL/L of 1000× vitamin stock, 0.1 mL/L of 10,000× mineral stock) supplemented with or without thiamine (10 μM) was used to culture and prepare *S. pombe* strains containing Tf1 reporter vectors for Tf1 retrotransposition assay.

### Construction of vectors and *S. pombe* strains for FnCpf1, LbCpf1 and LshCas13a expression

The codon-optimized *F. novicida* Cpf1 (FnCpf1) and *L. bacterium* Cpf1 (LbCpf1) tagged with C-terminal nuclear localization signal and 3× FLAG tag were PCR-amplified from plasmids FnCpf1-OsU6 and LbCpf1-OsU6 (gifts from Dr. Jiankang Zhu’s Lab) using the primer pair pHSN-Fn-*Nde*I-F and pHSN-Fn-*Nco*I-R (Supplementary Data S1). The plasmid pDUAL-HFF1 (RIKEN BioResource Center, Ibaraki, Japan) was digested with the *Nde*I/*Nco*I restriction enzymes, before it was assembled with the above amplified FnCpf1 or LbCpf1 PCR fragments, respectively, using ClonExpress® II One Step Cloning Kit (Vazyme), producing plasmids pDUAL-HFF1-FnCpf1 and pDUAL-HFF1-LbCpf1 (Supplementary Fig. S1 and Table S1). The pDUAL-HFF1-Cas13a expression vectors were generated in the previous study^47^. For chromosomal integration of FnCpf1 and LbCpf1 expression constructs, the plasmids pDUAL-HFF1-FnCpf1 and pDUAL-HFF1-LbCpf1 were digested with the *Not*I restriction enzyme, and treated with FastAP Thermosensitive Alkaline Phosphatase (Thermo Scientific, Shanghai) before linearized DNA was recovered from agarose gel electrophoresis for yeast transformation. Transformants with FnCpf1, or LbCpf1 expression constructs were selected on EMM plates (supplemented with 50 μg/mL uracil), and verified for chromosomal integration by PCR with primer set ADHterm-F and leu1-R^59^ (Supplementary Data S1). For episomal expression of FnCpf1 in *S. pombe*, plasmid pDUAL-HFF1-FnCpf1 was modified by adding five crRNAs targeting *MEL1* and one nontargeting crRNA to produce six plasmids (Supplementary Table S3), which were used to transform yeast cells. Transformants were selected on EMM plates (supplemented with 50 μg/mL leucine), and tested for *MEL1* gene editing on agar plates containing 60 μg/mL X-α-Gal.

### Measurement of growth rates for different *S. pombe* strains

Various *S. pombe* strains were first plated in EMM plates supplemented with uracil (50 μg/mL) and allowed to grow at 32 °C for several days. Colonies were picked to seed liquid cultures of 3 mL EMM (with appropriate supplements), which were grown to mid-log phase. Cells were harvested and used to inoculate 20 mL EMM (with appropriate supplements) in shake-flask culture with an initial optical density (OD_600_) of 0.05 (or indicated otherwise). Growth of each culture was monitored by measuring OD_600_ at constant intervals using Thermo Scientific NanoDrop 2000c spectrophotometer. The growth curves were plotted with at least four replicates of shake-flask cultures.

To detect any toxicity of FnCpf1 and LbCpf1 in *S. pombe* cells, the growth rates of *S. pombe* strains with genome-integrated FnCpf1 or LbCpf1 expression constructs were measured and compared with that of isogenic control strains (transformed with control plasmid). To determine whether activation of CRISPR-Cas13a by Tf1 transcripts induces cell growth arrest, the growth rates of LshCas13a-carrying *S. pombe* strains (for which LshCas13a was continually activated in the presence of Tf1 transcripts and crRNAs) in liquid culture media were measured.

### Construction of Tf1 retrotransposition reporters with artificial introns

Our Tf1 retrotransposition reporter plasmids were constructed from pHL414 (a gift from Dr. Levin lab), based on the scheme from the previous study^69^. The plasmid pHL414 carrying Tf1 retrotransposon was modified by inserting an artificial intron sequence into the ORF of *NEO* gene in opposite orientation to the *NEO* transcript, which would align in the same orientation to that of Tf1 (Fig. 2a). Four different sites with nearby PAM sequences in the *NEO* gene were selected for intron insertion (Fig. 2b).

To construct the four reporters with intron, *NEO* fragments (~250 bp) (Supplementary Data S1) with artificial introns inserted in the above four different sites selected near PAM sequences, were first synthesized by Genwiz Biotech (Suzhou, China). These *NEO* fragments were PCR-amplified using the primer set Neo-TYCZ-P5 and Neo-TYCZ-P3 (Supplementary Data S1), and were inserted into linearized pHL414 (digested with *Eco*NI/*Nru*I) by recombination using ClonExpress® II One Step Cloning Kit. The series of Tf1 retrotransposition reporters with artificial introns are included in Supplementary Table S4.

### Construction of crRNA vectors to direct targeting by FnCpf1, LbCpf1, and LshCas13a

Construction of crRNA expression plasmids for *S. pombe* was similar to those of the previous study^47^. Briefly, crRNA-backbone plasmids were first generated in plasmid pBluescript II KS for FnCpf1, LbCpf1, and LshCas13a, respectively (Supplementary Table S1), comprising the *rrk1* promoter, direct repeat sequence (DR), *Bsp*QI placeholder for spacer sequence, and HDVR (hepatitis delta virus ribozyme)^34,62^. The optimized DR lengths for FnCpf1, LbCpf1, and LshCas13a are 20, 21, and 28 bp, respectively, whereas lengths for their spacer sequences are 25, 25, and 28 bp, respectively. Then, an intermediate construct (Supplementary Table S2) for each crRNA cassette was completed, using the oligonucleotide pairs designed for spacer of each crRNA (Supplementary Data S1). The oligonucleotide pairs were first annealed to form stick-end, before being cloned into the *Bsp*QI placeholder.

For generation of FnCpf1 and LbCpf1 crRNAs to target the endogenous *MEL1* gene, the corresponding intermediate constructs were used as templates to PCR-amplify the crRNA cassettes (comprising *rrk1* promoter, DR, spacer and HDVR), using the primer set pDUAL-*Spe*I-T3 and pDUAL-PspXI-T7 (Supplementary Data S1). The crRNA cassette PCR fragments were then inserted into linearized plasmids pDUAL-HFF1 (digested with *Spe*I/*Xho*I) by recombination using ClonExpress® II One Step Cloning Kit (Vazyme). As for crRNAs for episomal expression of FnCpf1, the crRNA cassette fragments were inserted into the FnCpf1 expression plasmid pDUAL-HFF1-FnCpf1, instead of pDUAL-HFF1 (Supplementary Table S3).

For generation of FnCpf1 crRNAs to target the four *NEO* sequences (generated via splicing) within Tf1 DNA intermediates (Fig. 2b), the corresponding intermediate constructs were used as templates to PCR-amplify the crRNA cassettes, using the primer set pHL414-*Nhe*I-T7 and pHL414-NheI-T3 (Supplementary Data S1). The crRNA cassette PCR fragments were then inserted into linearized plasmids of Tf1 retrotransposition reporters (digested with *Nhe*I) to form nine crRNA vectors (Supplementary Table S4), by recombination using ClonExpress® II One Step Cloning Kit (Vazyme), respectively.

For generation of genome-integrated FnCpf1 crRNA constructs to target the four *NEO* sequences (generated via splicing) within Tf1 DNA intermediates (Fig. 2b), the corresponding intermediate constructs were used as templates to PCR-amplify the crRNA cassettes, using the primer set pDUAL-SpeI-SK and pDUAL-SpeI-T3 (Supplementary Data S1). The crRNA cassette PCR fragments were then inserted into linearized FnCpf1 expression plasmid, pDUAL-HFF1-FnCpf1 (digested with *Spe*I) to form six crRNA vectors (Supplementary Table S4), by recombination using ClonExpress® II One Step Cloning Kit (Vazyme). The resulting six plasmids (five targeting crRNA and one nontargeting control) were linearized by *Not*I digestion for yeast transformation to create six different genome-integrated crRNA construct strains.

For generation of LshCas13a crRNAs to target Tf1 RNA intermediates, the corresponding intermediate constructs were used as templates to PCR-amplify the crRNA cassettes, using the primer set *Nhe*I-TYB-P5 and *Nhe*I-TYB-P3 (Supplementary Data S1). The crRNA cassette PCR fragments were then inserted into linearized plasmid pHL414 carrying Tf1 retrotransposon (digested with *Nhe*I) to form three crRNA vectors (Supplementary Table S5), by recombination using ClonExpress® II One Step Cloning Kit (Vazyme).

In above crRNA constructs, a nontargeting crRNA was designed and constructed as Cas12a and Cas13a targeting controls, respectively. For Cas12a, the nontargeting spacer sequence was “AACAGCGCCTTAAAAGAACTAGAAA”. For Cas13a, the nontargeting spacer sequence was “CAGACTATGCGTCGACAAGCCAGGCATT”. All constructed plasmids were validated by diagnostic PCR and Sanger sequencing.

### Assay for Tf1 retrotransposition in *S. pombe*

To qualitatively determine the Tf1 retrotransposition, the “transfer-and-patch” assay was performed following the previously developed protocol^47,69^. In brief, various *S. pombe* strains were first plated in EMM plates supplemented with thiamine (10 μM) and were grown for 4 days at 32 °C. Then individual colonies were picked with sterile toothpicks and transferred on PMG plates with thiamine (10 μM) for patch growth. After incubation at 32 °C for 3 days, the patches were transferred to PMG plates (without thiamine to initiate transcription and retrotransposition), and allowed to grow for 4 days. The patches were transferred to PMG plates containing 5-FOA, uracil and thiamine, and allowed to grow for 3 days before patches were transferred to YES plates containing 5-FOA, uracil and G418.

To quantitatively measure Tf1 retrotransposition frequency, the colony-forming assay was performed following the previously developed protocol^47,69^. In brief, various *S. pombe* strains were first plated on EMM plates supplemented with thiamine (10 μM) and were grown for 4 days at 32 °C. Then individual colonies were picked and transferred on PMG plates with thiamine (10 μM) for patch growth. After incubation at 32 °C for 3 days, the patches were transferred to liquid PMG medium (without thiamine to initiate transcription and retrotransposition) at OD_600_ of 0.05. The cultures were allowed to grow at 32 °C for another 4 days before being treated in liquid PMG containing 5-FOA (1 mg/mL) and uracil (50 μg/mL) for 36 h. The cultures were used subsequently to make serial dilutions for 10^7^, 10^6^ and 10^5^ cells/mL, and 100 μL of them were plated onto paired YES plates containing either 5-FOA or both 5-FOA and G418. Plated cells were allowed to grow for 2−3 days, and the numbers of colonies formed on paired plates were counted, for which frequencies of Tf1 retrotransposition were estimated by comparing the colony numbers from paired plates with both G418 and 5-FOA, or with 5-FOA only.

## Supplementary information

Supplementary information

## Supplementary information

**Supplementary Information** accompanies the paper at (10.1038/s41421-020-0164-0).
