# Supplementary material for: Interfering with retrotransposition by two types of CRISPR effectors: Cas12a and Cas13a
Source: Cell Discov. 2020 May 19;6:30. doi: 10.1038/s41421-020-0164-0 (PMC7235257; doi:10.1038/s41421-020-0164-0)
Supplement: Supplementary file 1 — Supplementary information [file 41421_2020_164_MOESM1_ESM.pdf]

## Supplementary Information for

# Interfering with retrotransposition by two types of CRISPR effectors: Cas12a and Cas13a

Niubing Zhang[1,3,#], Xinyun Jing[1,#], Yuanhua Liu[2], Minjie Chen[1,3], Xianfeng Zhu[3], Jing Jiang[3], Hongbing Wang[4], Xuan Li[1,*], and Pei Hao[2,*]

# CONTENTS:

Supplementary Figures,

Supplementary Tables,

Supplementary Data S1,

Supplementary Data S2.

## SUPPLEMENTARY FIGURES

### Figure S1

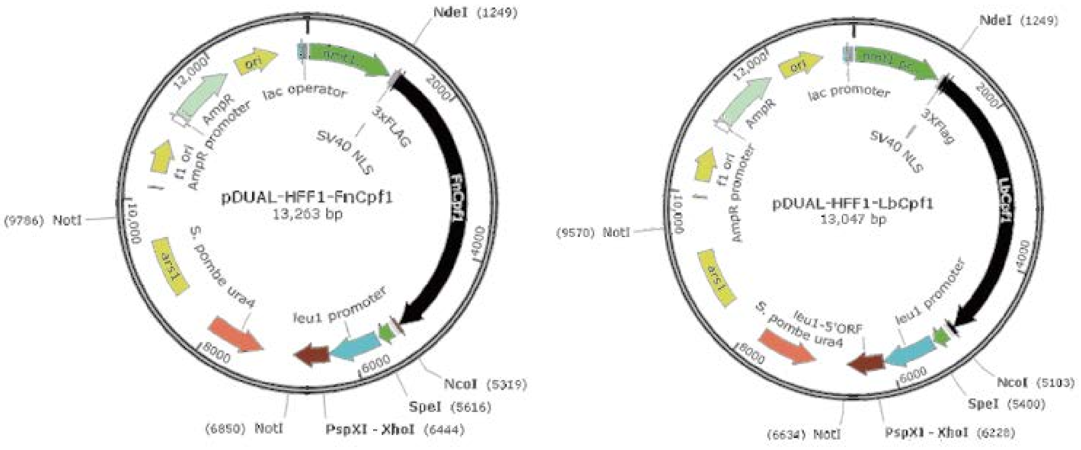


**Figure S1. Maps of plasmids of pDUAL-HFF1-FnCpf1 and pDUAL-HFF1-LbCpf1 for chromosome-integrated expression of FnCpf1 and LbCpf1 in *S. pombe*.** The FnCpf1 and LbCpf1 expression cassettes contain the *nmt*1 promoter and the *ADH*1 terminator. The SV40-derived nuclear localization signal (NLS) was fused to both N and C termini of Cpf1; a 3 × Flag was in-frame fused to the N terminus of NLS.

# Figure S2

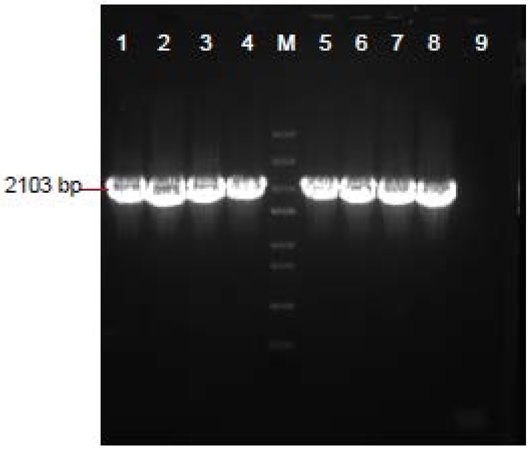


**Figure S2. Diagnostic PCR for *S. pombe* transformants with FnCpf1 and LbCpf1 expression cassettes integrated into the chromosome.** Primer set ADHterm-F/leu1-R (Supplementary Data 1) was used for diagnostic PCR. Lanes 1-4: *S. pombe* transformants with integrated expression construct of FnCpf1; Lanes 5-8: *S. pombe* transformants with integrated expression construct of LbCpf1; Lane 9: control wild-type *S. pombe* strain (without integrated expression constructs); M: *Trans*5K DNA Marker (Trans Transgen Biotech, Shanghai, China).

# Figure S3

a

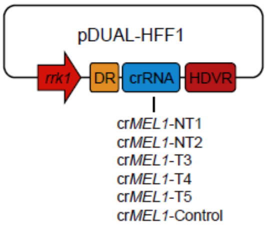


b

| Target gene | crRNA name | PAM - guide sequence (5'-3') | Position from ATG | Gene editing efficiency | |
|---|---|---|---|---|---|
| | | | | FnCpf1 chromosomal | LbCpf1 chromosomal |
| *MEL1* (1311 nt) | cr*MEL1*-NT1 | T(TTG)CAGATTGGGGAGTAGATTACTTAAA | 431 | 71% | 28% |
| | cr*MEL1*-NT2 | T(TTC)CAATAATGATTGGGTAGTGGCTGTT | 1050 | 29% | 20% |
| | cr*MEL1*-T3 | T(TTA)GTCCCAAACCATTATAGCTACCATG | 67 | 77% | 14% |
| | cr*MEL1*-T4 | T(TTG)CTTCCAATCTACCAGTGGTTGCATT | 250 | 45% | 35% |
| | cr*MEL1*-T5 | T(TTA)AGTAATCTACTCCCCAATCTGCAAA | 427 | 77% | 53% |
| | cr*MEL1*-Control | AACAGCGCCTTAAAAGAACTAGAAA | | 0 | 0 |

**Figure S3. Editing on *MEL1* gene by chromosomal expressing FnCpf1 and LbCpf1 with different crRNAs.** (a) Schematic representation the crRNA expression cassette for FnCpf1 and LbCpf1. *rrk1*, *rrk1* promoter; DR, direct repeat of crRNA; HDVR, hepatitis delta virus ribozyme. (b) Targeting sequences and editing efficiency on *MEL1* gene by chromosomal expressing FnCpf1 or LbCpf1 with five different crRNAs in *S.pombe*. The PAM motif (TTTN for FnCpf1 and LbCpf1) regions are underlined.

# Figure S4

a

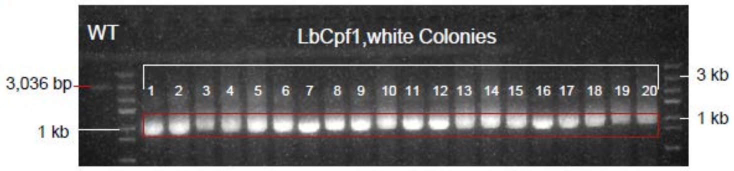


**b**

**cr*MEL1*-T5 _Col1:**

TTCTTATACGGAATACATACAATTATATGCACTGAAAAGTTTTGCCACATTCGCCTCCGTAGTACTTCAAAATGCCAATAGTAA
TTTACATTTTACAAGTTGAAAAACAAAAATGGCTGTAGTACTTGTTACTCATTTAAAGCTTTCAAGTTACAATTTTTTAGAATC
AGATCAGGCAGACTGCTGGCAAAGGGCAAAACTAATGCCTATTATATCATCTGACGCACTTGGAGCTGTACCACGTTTGGTT
AGCTTCCCATTTTATTGAATACTGAGAATTGCAACAATCTCTTATATTTATAATGCTCGATGTCCTGTTAACGAAAAATAAAGT
ATTTAAATGGATTGAGTAAAAATGAACCGTAAAGATTTGTAATGTATTACCGTAGCACATGAGTTAAATAATCCGCATTCCAA
TATGTCCTTGTAAAAATCTTATTAAGTAACCGTAACGTTTTATCATTATGTAAGCGATATTGACTTCTTGTTC
CCAGAATCGACCTAGCACATGATATTTTTTTTCGAATAAATTCGAATATAAAAAAAATTTTACGTATGTAACATAATTTGAATG
CTCTTACCAAAAACCCCGATACAGATCATAATGCCTTTTTTTATTTTTTTAAACATTTTTGCGCCTTTCCGGTTATTGTCAAAATT
CGAAAAATTTTTTTAAAAAAGTTTATTATTATTTTTTTCTGTACATCAAAATATTCAACGCTAAGAAAACCATAGAAAAATGCG
CATCAAAATTTTAATTAGGATATAGCAGATGCTATAACAAAAGGATTTGTCTAAAACCTAGAGATATTATTAAAGCTTTTCTAA
TTGCACGCCAGTTTATATATCATTTAATGATTTTCATTTTCTGCTAGAAATAGTTATATTT

**cr*MEL1*-T5 _Col2:**

TTCTTATACGGAATACATACAATTATATGCACTGAAAAGTTTTGCCACATTCGCCTCCGTAGTACTTCAAAATGCCAATAGTAA
TTTACATTTTACAAGTTGAAAAACAAAAATGGCTGTAGTACTTGTTACTCATTTAAAGCTTTCAAGTTACAATTTTTTAGAATC
AGATCAGGCAGACTGCTGGCAAAGGGCAAAACTAATGCCTATTATATCATCTGACGCACTTGGAGCTGTACCACGTTTGGTT
AGCTTCCCATTTTATTGAATACTGAGAATTGCAACAATCTCTTATATTTATAATGCTCGATGTCCTGTTAACGAAAAATAAAGT
ATTTAAATGGATTGAGTAAAAATGAACCGTAAAGATTTGTAATGTATTACCGTAGCACATGAGTTAAATAATCCGCATTCCAA
TATGTCCTTGTAAAAATCTTATTAAGTAACCGTAACGTTTTATCATTATGTAAGCGATATTGACTTCTTGTTC
CCAGAATCGACCTAGCACATGATATTTTTTTTCGAATAAATTCGAATATAAAAAAAATTTTACGTATGTAACATAATTTGAATG
CTCTTACCAAAAACCCCGATACAGATCATAATGCCTTTTTTTATTTTTTTAAACATTTTTGCGCCTTTCCGGTTATTGTCAAAATT
CGAAAAATTTTTTTAAAAAAGTTTATTATTATTTTTTTCTGTACATCAAAATATTCAACGCTAAGAAAACCATAGAAAAATGCG
CATCAAAATTTTAATTAAGATATAGCAGATGCTATAACAAAAGGATTTGTCTAAAACCTAGAGATATTATTAAAGCTTTTTCTA
ATTGCACGCCAGTTT

**c**

AGGTTAAAACTCCAGAACATTACTTAATCAAAAAATATGCTGTGGTCGAGAAATTCTTATACGGAATACATACAATTATATGCACT
GAAAAGTTTTGCCACATTCGCCTCCGTAGTACTTCAAAATGCCAATAGTAATTTACATTTTACAAGTTGAAAAACAAAAATGGCT
GTAGTACTTGTTACTCATTTAAAGCTTTCAAGTTACAATTTTTTAGAATCAGATCAGGCAGACTGCTGGCAAAGGGCAAAACTAA
TGCCTATTATATCATCTGACGCACTTGGAGCTGTACCACGTTTGGTTAGCTTCCCATTTTATTGAATACTGAGAATTGCAACAATCT
CTTATATTTATAATGCTCGATGTCCTGTTAACGAAAAATAAAGTATTTAAATGGATTGAGTAAAAATGAACCGTAAAGATTTGTAAT
GTATTACCGTAGCACATGAGTTAAATAATCCGCATTCCAATATGTCCTTGTAAAAATCTTATTAAGTAACCGTAACGTTTTATCATTA
TGTAAGCGATATTGACTTCTTGTTCCCAGAATCGACCTAGCACATGATATTTTTTTTCGAATAAATTCGAATATAAAAAAAATTTTA

CGTATGTAACATAATTTGAATGCTCTTACCAAAAACCCCGATACAGATCATAATGCCTTTTTTTATTTTTTTAAACATTTTTGCGCCT
TTCCGGTTATTGTCAAAATTCGAAAAATTTTTTTAAAAAAGTTTATTATTATTTTTTTCTGTACATCAAAATATTCAACGCTAAGAA
AACCATAGAAAAATGCGCATCAAAATTTTAATTAAGATATAGCAGATGCTATAACAAAAGGATTTGTCTAAAACCTAGAGATATTA
TTAAAGCTTTTTCTAATTGCACGCCAGTTTTATATATCATTTAATGATTTTCATTTTTCTGTCTAGTAAATAGTTATATTTTGATTGATA
ACTAACAAATTTTATTAAATTCGGGGCTCGTTCTACTATAGC

**Figure S4. Diagnostic PCR products and sequencing of *mel1* mutants generated by LbCpf1 editing.** (a) Diagnostic PCR products of white colonies of *mel1* mutants generated by LbCpf1. All colonies show a 998-bp band indicating deletion of *MEL1* from LbCpf1 editing. The wild type strain shows a 3036-bp band. The primer set used for PCR were described in Figure 1c. (b) Sequences of PCR products from two randomly selected *mel1* mutant colonies. The sequence results show the correct deletion of the center portion of *MEL1* sequence. The blue and yellow highlighted sequences represent the upstream and downstream regions of *MEL1* gene, whereas the central potion is deleted. (c) Sequence of the template which was used for editing of *MEL1* gene by FnCpf1 or LbCpf1 through homologous recombination (HR). Blue sequence, the left arm of the HR template, which was amplified using primers of MEL1-P5, mel1-left-p3-new from *S.pombe* genomic DNA. Yellow sequence, the right arm of the HR template, which was amplified using primers MEL1-P3 and mel1-right-P5-new from *S.pombe* genomic DNA. The HR template was obtained by over-lap PCR using primers MEL1-P5, MEL1-P3 from left and right arms.

# Figure S5

a

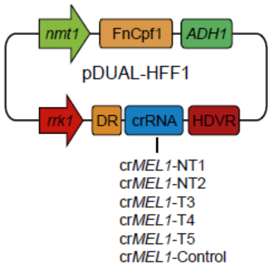


b

| Target gene | crRNA name | PAM - guide sequence (5'-3') | Position from ATG | Gene editing efficiency | |
|---|---|---|---|---|---|
| | | | | FnCpf1 chromosomal | FnCpf1 episomal |
| *MEL1* (1311 nt) | cr*MEL1*-NT1 | T(TTG)CAGATTGGGGAGTAGATTACTTAAA | 431 | 71% | 29% |
| | cr*MEL1*-NT2 | T(TTC)CAATAATGATTGGGTAGTGGCTGTT | 1050 | 29% | 59% |
| | cr*MEL1*-T3 | T(TTA)GTCCCAAACCATTATAGCTACCATG | 67 | 77% | 67% |
| | cr*MEL1*-T4 | T(TTG)CTTCCAATCTACCAGTGGTTGCATT | 250 | 45% | 38% |
| | cr*MEL1*-T5 | T(TTA)AGTAATCTACTCCCCAATCTGCAAA | 427 | 77% | 46% |
| | cr*MEL1*-Control | AACAGCGCCTTAAAAGAACTAGAAA | | 0 | 0 |

**Figure S5. Editing efficiency on *MEL1* gene by chromosomal-expressing FnCpf1 and episomal-expressing FnCpf1 with five different crRNAs.** (a) Schematic representation of the FnCpf1 and crRNA expression cassette in multi-copy plasmid pDUAL-HFF1. *rrk1*, *rrk1* promoter; DR, direct repeat of crRNA; HDVR, hepatitis delta virus ribozyme. (b) Targeting sequences and editing efficiency on *MEL1* gene by chromosomal-expressing FnCpf1 and episomal-expressing FnCpf1 with five different crRNAs in *S.pombe*. The PAM motif (TTTN for FnCpf1) regions are underlined.

# Figure S6

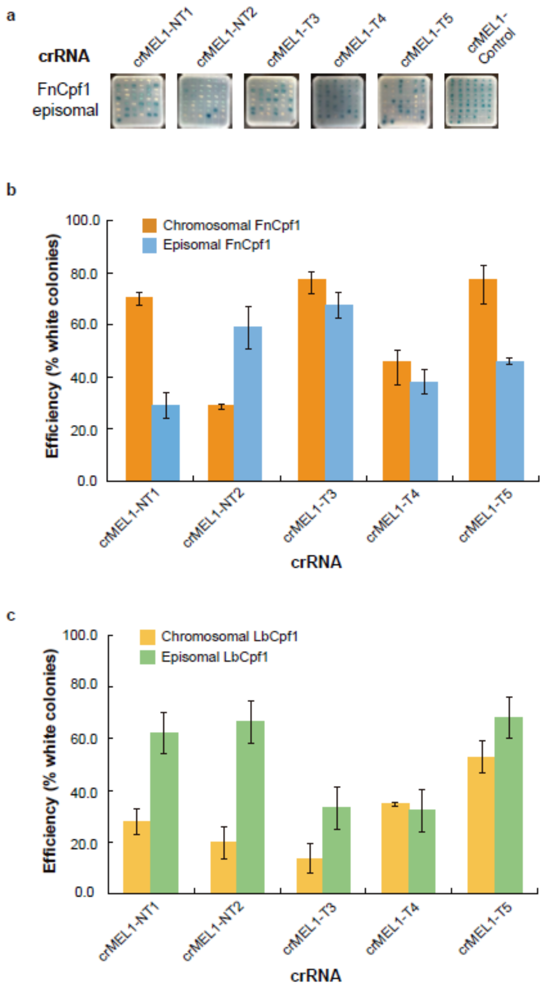


**Figure S6. Editing of *MEL1* gene by chromosomal-expressing FnCpf1 and episomal-expressing FnCpf1 with five different crRNAs.** (a) The editing on *MEL1* gene by episomal-expressing FnCpf1 with five different crRNAs, estimated by formation of white- (*mel1* mutant) and blue-colored (*MEL1* wildtype) colonies, assayed on agar plates containing the X-α-Gal. (b) Efficiency of genome editing on *MEL1* gene by chromosomal expressing FnCpf1 and episomal expressing FnCpf1 with five different crRNAs. Bar represents means ± s.e.m. with n = 3. (c) Efficiency of genome editing on *MEL1* gene by chromosomal expressing LbCpf1 and episomal expressing LbCpf1 with five different crRNAs. Bar represents means ± s.e.m. with n = 3.

# Figure S7

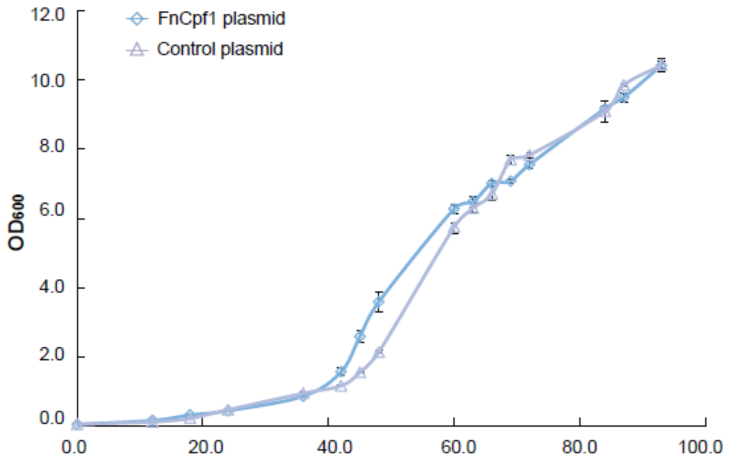


**Figure S7. Growth in liquid culture media (EMM + uracil) of *S. pombe* strains with episomal expressing FnCpf1 compared to the control strain with control plasmid pDUAL-HFF1.** They had an average double time of 9.2 h and 9.6 h, respectively. Data are means ± s.e.m. (n = 4 independent experiments)

# Figure S8

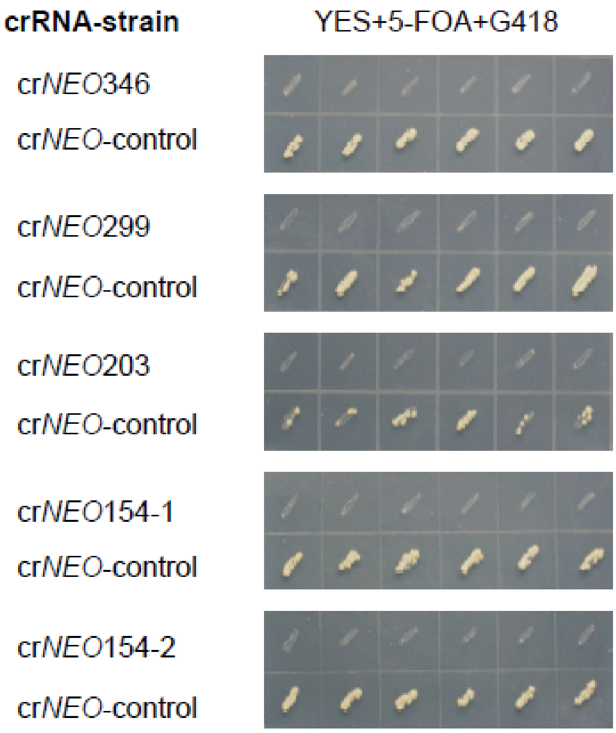


**Figure S8. Prolonged crRNA targeting eliminating residual Tf1 retrotransposition by Cas12a.**
‘Transfer-and-patch’ assay for detection of Tf1 retrotransposition using plates containing YES media with 5-FOA and G418. The effects of persistent crRNA targeting in their genome-integrated strains were compared to crNEO-control strains side by side.

## Figure S9

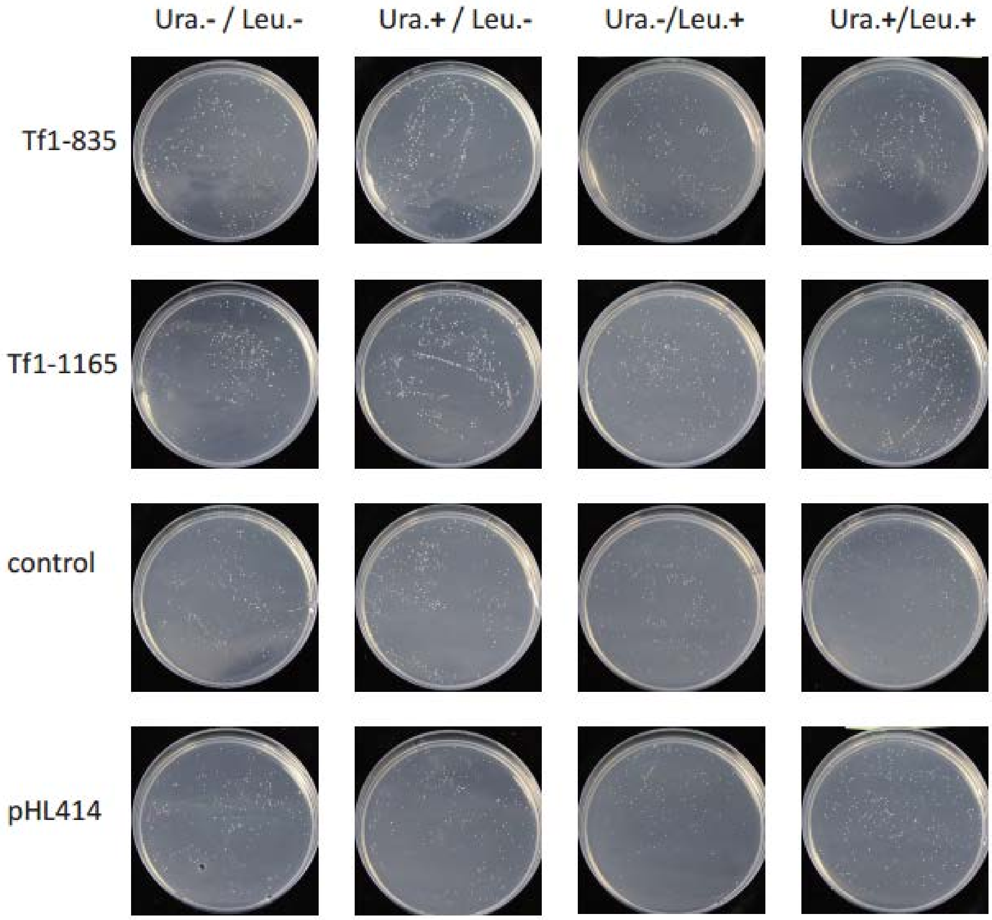


**Figure S9. The ending *S. pombe* populations from one batch of liquid cultures (Fig. 5c) were further checked for presence of selection markers for Cas13a gene (Leu+) and Tf1/crRNA-carrying plasmid (Ura+)**

100 μL of 1000-fold diluted cells at late log-phase cultures (Fig. 5c) were plated to four different EMM selective plates (uracil-/leucine-, uracil+/leucine-, uracil-/leucine+, and uracil+/leucine+) respectively. The presence of Cas13a gene and Tf1/crRNA-carrying plasmid were validated by colony-formation on selective media plates.

# SUPPLEMENTARY TABLES

**Supplementary Table S1.** List of strains and plasmids used in this study.

| Strains and plasmids | Characteristics | Source/Reference |
|---|---|---|
| **Strains** | | |
| *E.coli* DH5α | *F- eNDA1 glnV44 thi-1 recA1 relA1 gyrA96 deoRnupG Φ80dlacZΔM15 Δ(lacZYA-argF)U169, hsdR17 (rK-mK), λ–* | Takara Biotechnology Co.,Ltd. |
| *S.pombe* FY7652 | *h- leu1-32 ura4-D18* | National Bio Resource Project |
| **Plasmids** | | |
| FnCpf1-OsU6 | *E.coli , pBR322 ori* , $Kan^r$, FnCpf1, crRNA cassette for expression in rice | Gift from Dr. Jiankang Zhu's Lab[1] |
| LbCpf1-OsU6 | *E.coli , pBR322 ori* , $Kan^r$, LbCpf1, crRNA cassette for expression in rice | Gift from Dr. Jiankang Zhu's Lab[1] |
| pDUAL-HFF1 | *E.coli -S.pombe* shutting vector, *ars1 ori, ColE1 ori, f1 ori* $Amp^r$ , *ura4* | RIKEN BRC (RDB:6179) |
| pDUAL-HFF1-FnCpf1 | *FY7652*, FnCpf1/*nmt1* | This study |
| pDUAL-HFF1-LbCpf1 | *FY7652*, LbCpf1/*nmt1* | This study |
| pDUAL-HFF1-Cas13a | *FY7652*, LshCas13a/*nmt1* | Our Lab[2] |
| pHL414 | *S. pombe*, Tf1 retrotransposon/*nmt1* | Gift from Professor Henry L. Levin's lab[3] |
| pBluescript II KS(+) | *E.coli , ColE1 ori, f1 ori*, $Amp^r$ | |
| pKS-rrk1-FnCpf1-crRNA-backbone | *E.coli,* crRNA backbone plasmid for FnCpf1, $Amp^r$ | This study |
| pKS-rrk1-LbCpf1-crRNA-backbone | *E.coli,* crRNA backbone plasmid for LbCpf1, $Amp^r$ | This study |
| pKS-rrk1-LshCas13a-crRNA-backbone | *E.coli,* crRNA backbone plasmid for LshCas13a, $Amp^r$ | This study |

**Supplementary Table S2.** Intermediate plasmids for crRNA constructs.

| **Intermediate plasmids** | **Primers** | **Cloning sites** | **Original plasmids** | **Source** |
|---|---|---|---|---|
| pKS-rrk1-(FnCpf1 cr*MEL1*)-NT1 | crMEL1-NT1-P5<br>crMEL1-NT1-P3 | *BspQ*I | pKS-rrk1-FnCpf1-crRNA-backbone | This study |
| pKS-rrk1-(FnCpf1 cr*MEL1*)-NT2 | crMEL1-NT2-P5<br>crMEL1-NT2-P3 | *BspQ*I | pKS-rrk1-FnCpf1-crRNA-backbone | This study |
| pKS-rrk1-(FnCpf1 cr*MEL1*)-T3 | crMEL1-T3-P5<br>crMEL1-T3-P3 | *BspQ*I | pKS-rrk1-FnCpf1-crRNA-backbone | This study |
| pKS-rrk1-(FnCpf1 cr*MEL1*)-T4 | crMEL1-T4-P5<br>crMEL1-T4-P3 | *BspQ*I | pKS-rrk1-FnCpf1-crRNA-backbone | This study |
| pKS-rrk1-(FnCpf1 cr*MEL1*)-T5 | crMEL1-T5-P5<br>crMEL1-T5-P3 | *BspQ*I | pKS-rrk1-FnCpf1-crRNA-backbone | This study |
| pKS-rrk1-(FnCpf1 cr*MEL1*)-control | crMEL1-Control-P5<br>crMEL1-Control-P3 | *BspQ*I | pKS-rrk1-FnCpf1-crRNA-backbone | This study |
| pKS-rrk1-(LbCpf1 cr*MEL1*)-NT1 | crMEL1-NT1-P5<br>crMEL1-NT1-P3 | *BspQ*I | pKS-rrk1-LbCpf1-crRNA-backbone | This study |
| pKS-rrk1-(LbCpf1 cr*MEL1*)-NT2 | crMEL1-NT2-P5<br>crMEL1-NT2-P3 | *BspQ*I | pKS-rrk1-LbCpf1-crRNA-backbone | This study |
| pKS-rrk1-(LbCpf1 cr*MEL1*)-T3 | crMEL1-T3-P5<br>crMEL1-T3-P3 | *BspQ*I | pKS-rrk1-LbCpf1-crRNA-backbone | This study |
| pKS-rrk1-(LbCpf1 cr*MEL1*)-T4 | crMEL1-T4-P5<br>crMEL1-T4-P3 | *BspQ*I | pKS-rrk1-LbCpf1-crRNA-backbone | This study |
| pKS-rrk1-(LbCpf1 cr*MEL1*)-T5 | crMEL1-T5-P5<br>crMEL1-T5-P3 | *BspQ*I | pKS-rrk1-LbCpf1-crRNA-backbone | This study |
| pKS-rrk1-(LbCpf1 cr*MEL1*)-control | crMEL1-Control-P5<br>crMEL1-Control-P3 | *BspQ*I | pKS-rrk1-LbCpf1-crRNA-backbone | This study |
| pKS-rrk1-(FnCpf1 cr*NEO*346) | crNEO346-P5<br>crNEO346-P3 | *BspQ*I | pKS-rrk1-FnCpf1-crRNA-backbone | This study |
| pKS-rrk1-(FnCpf1 cr*NEO*299) | crNEO299-P5<br>crNEO299-P3 | *BspQ*I | pKS-rrk1-FnCpf1-crRNA-backbone | This study |
| pKS-rrk1-(FnCpf1 cr*NEO*203) | crNEO203-P5<br>crNEO203-P3 | *BspQ*I | pKS-rrk1-FnCpf1-crRNA-backbone | This study |
| pKS-rrk1-(FnCpf1 cr*NEO*154-1) | crNEO154-1-P5<br>crNEO154-1-P3 | *BspQ*I | pKS-rrk1-FnCpf1-crRNA-backbone | This study |
| pKS-rrk1-(FnCpf1 cr*NEO*154-2) | crNEO154-2--P5<br>crNEO154-2-P3 | *BspQ*I | pKS-rrk1-FnCpf1-crRNA-backbone | This study |
| pKS-rrk1-(FnCpf1 cr*NEO*-Control) | crNEO-Control-P5<br>crNEO-Control-P3 | *BspQ*I | pKS-rrk1-FnCpf1-crRNA-backbone | This study |
| pKS-rrk1-(LshCas13a crRNA)-(Tf1-835) | Tf1-835-gRNA-P5<br>Tf1-835-gRNA-P3 | *BspQ*I | pKS-rrk1-LshCas13a-crRNA-backbone | This study |
| pKS-rrk1-(LshCas13a crRNA)-(Tf1-1165) | Tf1-1165-gRNA-P5<br>Tf1-1165-gRNA-P3 | *BspQ*I | pKS-rrk1-LshCas13a-crRNA-backbone | This study |
| pKS-rrk1-(LshCas13a crRNA)-Control | Tf1-Control-gRNA-P5<br>Tf1-Control-gRNA-P3 | *BspQ*I | pKS-rrk1-LshCas13a-crRNA-backbone | This study |

**Supplementary Table S3.** crRNA Constructs for targeting the endogenous *MEL1* gene by FnCpf1 and LbCpf1.

| **Plasmid Name** | **Genes/Promoters** | **crRNA primers** | **Original plasmid** | **Descriptions** | **Source** |
|---|---|---|---|---|---|
| pDUAL-HFF1-(*Fn*Cpf1 cr*MEL1*)-NT1 | crRNA/*rrk1* | crMEL1-NT1-P5 crMEL1-NT1-P3 | pDUAL-HFF1 | for chromosomal expression of FnCpf1 and episomal expression crRNA | This study |
| pDUAL-HFF1-(*Fn*Cpf1 cr*MEL1*)-NT2 | crRNA/*rrk1* | crMEL1-NT2-P5 crMEL1-NT2-P3 | pDUAL-HFF1 | for chromosomal expression of FnCpf1 and episomal expression crRNA | This study |
| pDUAL-HFF1-(*Fn*Cpf1 cr*MEL1*)-T3 | crRNA/*rrk1* | crMEL1-T3-P5 crMEL1-T3-P3 | pDUAL-HFF1 | for chromosomal expression of FnCpf1 and episomal expression crRNA | This study |
| pDUAL-HFF1-(*Fn*Cpf1 cr*MEL1*)-T4 | crRNA/*rrk1* | crMEL1-T4-P5 crMEL1-T4-P3 | pDUAL-HFF1 | for chromosomal expression of FnCpf1 and episomal expression crRNA | This study |
| pDUAL-HFF1-(*Fn*Cpf1 cr*MEL1*)-T5 | crRNA/*rrk1* | crMEL1-T5-P5 crMEL1-T5-P3 | pDUAL-HFF1 | for chromosomal expression of FnCpf1 and episomal expression crRNA | This study |
| pDUAL-HFF1-(*Fn*Cpf1 cr*MEL1*)-control | crRNA/*rrk1* | crMEL1-Control-P5 crMEL1-Control-P3 | pDUAL-HFF1 | for chromosomal expression of FnCpf1 and episomal expression crRNA | This study |
| pDUAL-HFF1-(*Lb*Cpf1 cr*MEL1*)-NT1 | crRNA/*rrk1* | crMEL1-NT1-P5 crMEL1-NT1-P3 | pDUAL-HFF1 | for chromosomal expression of LbCpf1 and episomal expression crRNA | This study |
| pDUAL-HFF1-(*Lb*Cpf1 cr*MEL1*)-NT2 | crRNA/*rrk1* | crMEL1-NT2-P5 crMEL1-NT2-P3 | pDUAL-HFF1 | for chromosomal expression of LbCpf1 and episomal expression crRNA | This study |
| pDUAL-HFF1-(*Lb*Cpf1 cr*MEL1*)-T3 | crRNA/*rrk1* | crMEL1-T3-P5 crMEL1-T3-P3 | pDUAL-HFF1 | for chromosomal expression of LbCpf1 and episomal expression crRNA | This study |
| pDUAL-HFF1-(*Lb*Cpf1 cr*MEL1*)-T4 | crRNA/*rrk1* | crMEL1-T4-P5 crMEL1-T4-P3 | pDUAL-HFF1 | for chromosomal expression of LbCpf1 and episomal expression crRNA | This study |
| pDUAL-HFF1-(*Lb*Cpf1 cr*MEL1*)-T5 | crRNA/*rrk1* | crMEL1-T5-P5 crMEL1-T5-P3 | pDUAL-HFF1 | for chromosomal expression of LbCpf1 and episomal expression crRNA | This study |
| pDUAL-HFF1-(*Lb*Cpf1 cr*MEL1*)-control | crRNA/*rrk1* | crMEL1-Control-P5 crMEL1-Control-P3 | pDUAL-HFF1 | for chromosomal expression of LbCpf1 and episomal expression crRNA | This study |
| pDUAL-HFF1-*Fn*Cpf1-cr*MEL1*-NT1 | *Fn*Cpf1 / *nmt1* crRNA/*rrk1* | crMEL1-NT1-P5 crMEL1-NT1-P3 | pDUAL-FnCpf1-HFF1 | for episomal expression of FnCpf1 and crRNA | This study |
| pDUAL-HFF1-*Fn*Cpf1-cr*MEL1*-NT2 | *Fn*Cpf1 / *nmt1* crRNA/*rrk1* | crMEL1-NT2-P5 crMEL1-NT2-P3 | pDUAL-FnCpf1-HFF1 | for episomal expression of FnCpf1 and crRNA | This study |
| pDUAL-HFF1-*Fn*Cpf1-cr*MEL1*-T3 | *Fn*Cpf1 / *nmt1* crRNA/*rrk1* | crMEL1-T3-P5 crMEL1-T3-P3 | pDUAL-FnCpf1-HFF1 | for episomal expression of FnCpf1 and crRNA | This study |
| pDUAL-HFF1-*Fn*Cpf1-cr*MEL1*-T4 | *Fn*Cpf1 / *nmt1* crRNA/*rrk1* | crMEL1-T4-P5 crMEL1-T4-P3 | pDUAL-FnCpf1-HFF1 | for episomal expression of FnCpf1 and crRNA | This study |
| pDUAL-HFF1-*Fn*Cpf1-cr*MEL1*-T5 | *Fn*Cpf1 / *nmt1* crRNA/*rrk1* | crMEL1-T5-P5 crMEL1-T5-P3 | pDUAL-FnCpf1-HFF1 | for episomal expression of FnCpf1 and crRNA | This study |
| pDUAL-HFF1-*Fn*Cpf1-cr*MEL1*-control | *Fn*Cpf1 / *nmt1* crRNA/*rrk1* | crMEL1-Control-P5 crMEL1-Control-P3 | pDUAL-FnCpf1-HFF1 | for episomal expression of FnCpf1 and crRNA | This study |

**Supplementary Table S4.** crRNA constructs for targeting the Tf1 DNA intermediates (generated via splicing of artificial introns and reverse-transcription) by FnCpf1.

| Plasmid Name | Promoter | Original plasmid | Descriptions | Source |
|---|---|---|---|---|
| pHL414-Tf1-neo-intron346 | Tf1/*nmt1* | pHL414 | Tf1 retrotransposition reporters plasmid, artificial intron is 346bp from ATG of the NEO gene | This study |
| pHL414-Tf1-neo-intron299 | Tf1/*nmt1* | pHL414 | Tf1 retrotransposition reporters plasmid, artificial intron is 299bp from ATG of the NEO gene | This study |
| pHL414-Tf1-neo-intron203 | Tf1/*nmt1* | pHL414 | Tf1 retrotransposition reporters plasmid, artificial intron is 203bp from ATG of the NEO gene | This study |
| pHL414-Tf1-neo-intron154 | Tf1/*nmt1* | pHL414 | Tf1 retrotransposition reporters plasmid, artificial intron is 154bp from ATG of the NEO gene | This study |
| pHL414-Tf1-neo-intron346-(*Fn*Cpf1 crNEO346) | Tf1/*nmt1* crRNA/*rrk1* | pHL414-Tf1-neo -intron346 | for chromosomal expression of FnCpf1, episomal expression crRNA and Tf1 retrotransposition reporter | This study |
| pHL414-Tf1-neo-intron299-(*Fn*Cpf1 crNEO299) | Tf1/*nmt1* crRNA/*rrk1* | pHL414-Tf1-neo -intron299 | for chromosomal expression of FnCpf1, episomal expression crRNA and Tf1 retrotransposition reporter | This study |
| pHL414-Tf1-neo-intron203-(*Fn*Cpf1 crNEO203) | Tf1/*nmt1* crRNA/*rrk1* | pHL414-Tf1-neo -intron203 | for chromosomal expression of FnCpf1, episomal expression crRNA and Tf1 retrotransposition reporter | This study |
| pHL414-Tf1-neo-intron154-(*Fn*Cpf1 crNEO154-1) | Tf1/*nmt1* crRNA/*rrk1* | pHL414-Tf1-neo -intron154 | for chromosomal expression of FnCpf1, episomal expression crRNA and Tf1 retrotransposition reporter | This study |
| pHL414-Tf1-neo-intron154-(*Fn*Cpf1 crNEO154-2) | Tf1/*nmt1* crRNA/*rrk1* | pHL414-Tf1-neo -intron154 | for chromosomal expression of FnCpf1, episomal expression crRNA and Tf1 retrotransposition reporter | This study |
| pHL414-Tf1-neo-intron346-(FnCpf1 crNEO-Control) | Tf1/nmt1 crRNA/rrk1 | pHL414-Tf1-neo -intron346 | for chromosomal expression of FnCpf1, episomal expression crRNA and Tf1 retrotransposition reporter | This study |
| pHL414-Tf1-neo-intron299-(FnCpf1 crNEO-Control) | Tf1/nmt1 crRNA/rrk1 | pHL414-Tf1-neo -intron299 | for chromosomal expression of FnCpf1, episomal expression crRNA and Tf1 retrotransposition reporter | This study |
| pHL414-Tf1-neo-intron203-(FnCpf1 crNEO-Control) | Tf1/nmt1 crRNA/rrk1 | pHL414-Tf1-neo -intron203 | for chromosomal expression of FnCpf1, episomal expression crRNA and Tf1 retrotransposition reporter | This study |
| pHL414-Tf1-neo-intron154-(FnCpf1 crNEO-Control) | Tf1/nmt1 crRNA/rrk1 | pHL414-Tf1-neo -intron154 | for chromosomal expression of FnCpf1, episomal expression crRNA and Tf1 retrotransposition reporter | This study |
| pDUAL-HFF1-*Fn*Cpf1-cr*NEO*346 | *Fn*Cpf1 / *nmt1* crRNA/*rrk1* | pDUAL-FnCpf1-HFF1 | for chromosomal expression of FnCpf1 and crRNA; episomal expression Tf1 retrotransposition reporter | This study |
| pDUAL-HFF1-*Fn*Cpf1-cr*NEO*299 | *Fn*Cpf1 / *nmt1* crRNA/*rrk1* | pDUAL-FnCpf1-HFF1 | for chromosomal expression of FnCpf1 and crRNA; episomal expression Tf1 retrotransposition reporter | This study |
| pDUAL-HFF1-*Fn*Cpf1-cr*NEO*203 | *Fn*Cpf1 / *nmt1* crRNA/*rrk1* | pDUAL-FnCpf1-HFF1 | for chromosomal expression of FnCpf1 and crRNA; episomal expression Tf1 retrotransposition reporter | This study |
| pDUAL-HFF1-*Fn*Cpf1-cr*NEO*154-1 | *Fn*Cpf1 / *nmt1* crRNA/*rrk1* | pDUAL-FnCpf1-HFF1 | for chromosomal expression of FnCpf1 and crRNA; episomal expression Tf1 retrotransposition reporter | This study |
| pDUAL-HFF1-*Fn*Cpf1-cr*NEO*154-2 | *Fn*Cpf1 / *nmt1* crRNA/*rrk1* | pDUAL-FnCpf1-HFF1 | for chromosomal expression of FnCpf1 and crRNA; episomal expression Tf1 retrotransposition reporter | This study |
| pDUAL-HFF1-*Fn*Cpf1-cr*NEO*-control | *Fn*Cpf1 / *nmt1* crRNA/*rrk1* | pDUAL-FnCpf1-HFF1 | for chromosomal expression of FnCpf1 and crRNA; episomal expression Tf1 retrotransposition reporter | This study |

**Supplementary Table S5.** crRNA constructs for targeting the Tf1 RNA intermediates by LshCas13a.

| **Plasmid Name** | **Promoter** | **Original plasmid** | **target sequence (5' to 3')** | **Descriptions** | **Source** |
|---|---|---|---|---|---|
| pHL414-Tf1-(LshCas13a crRNA)-(Tf1-835) | Tf1/*nmt1* crRNA/*rrk1* | pHL414 | ATCCAACTAGGTTTAC CATTCTTCTTAA | for chromosomal expression of LshCas13a and episomal expression crRNA and Tf1 | This study |
| pHL414-Tf1-(LshCas13a crRNA)-(Tf1-1165) | Tf1/*nmt1* crRNA/*rrk1* | pHL414 | TGTCGTTCTCCTTTAAA AACTTATTGTT | for chromosomal expression of LshCas13a and episomal expression crRNA and Tf1 | This study |
| pHL414-Tf1-(LshCas13a crRNA)-Control | Tf1/*nmt1* crRNA/*rrk1* | pHL414 | AATGCCTGGCTTGTCG ACGCATAGTCTG | for chromosomal expression of LshCas13a and episomal expression crRNA and Tf1 | This study |

# References


1 Wang, M., Mao, Y., Lu, Y., Tao, X. & Zhu, J. K. Multiplex gene editing in rice using the CRISPR-Cpf1 system. *Mol. Plant* **10**, 1011-1013 (2017).

2 Jing, X. *et al.* Implementation of the CRISPR-Cas13a system in fission yeast and its repurposing for precise RNA editing. *Nucleic Acids Res.* **46**, 90 (2018).

3 Sangesland, M., Atwood-Moore, A., Rai, S. K. & Levin, H. L. Qualitative and quantitative assays of transposition and homologous recombination of the retrotransposon Tf1 in Schizosaccharomyces pombe. *Methods Mol. Biol.* **1400**, 117-130 (2016).

**Supplementary Data S1**

| Primers Name | Sequences (5'-3') | Purpose |
|---|---|---|
| **Construction of vectors and *S. pombe* strains for FnCpf1 and LbCpf1 expression** | | |
| pHSN-Fn-NdeI-F | GCATCACCACCATCAT CATATG gactataaggaccacg | Amplification of FnCpf1 and LbCpf1 |
| pHSN-Fn-NcoI-R | CATCGTCGTCCTTGTAGT CCATGG ttactttttctttttgcctg | Amplification of FnCpf1 and LbCpf1 |
| ADHterm-F | CTCTTATTGACCACACCTCTACC | Confirmation of correct integration FnCpf1 and LbCpf1 |
| leu1-R | GGTCATAAAGTTGAACGGATGTCG | Confirmation of correct integration FnCpf1 and LbCpf1 |
| **Construction of Tf1 retrotransposition reporters with artificial introns** | | |
| neo-intron299 | gaatgctgttttcccggggatcgGTAGGTGCTATTTTACTAGTCTAAGCTAATCAATAGcagtggtgagtaaccatgcatcatcaggagtacggataaaatgcttgatggtcggaagaggcataaattccgtcagccagtttagtctgaccatctcatctgtaacatcattggcaacgctacctttgccatgtttcagaaacaactctggcgcatcgggcttcccatacaatcgatagattgtcgcacct | To generate the NEO fragments with artificial intron |
| neo-intron203 | gaatgctgttttcccggggatcgcagtggtgagtaaccatgcatcatcaggagtacggataaaatgcttgatggtcggaagaggcataaattccgtcagccagtttagtctgaccatctGTAGGTGCTATTTTACTAGTCTAAGCTAATCAATAGcatctgtaacatcattggcaacgctacctttgccatgtttcagaaacaactctggcgcatcgggcttcccatacaatcgatagattgtcgcacct | To generate the NEO fragments with artificial intron |
| neo-intron154 | gaatgctgttttcccggggatcgcagtggtgagtaaccatgcatcatcaggagtacggataaaatgcttgatggtcggaagaggcataaattccgtcagccagtttagtctgaccatctcatctgtaacatcattggcaacgctacctttgccatgtttcagaaacaaGTAGGTGCTATTTTACTAGTCTAAGCTAATCAATAGctctggcgcatcgggcttcccatacaatcgatagattgtcgcacct | To generate the NEO fragments with artificial intron |
| neo-intron346-P5 | tcaacaatattttcacctgaGTAGGTGCTATTTTACTAGTCTAAGCTAATCAATAGatcaggatattcttctaatac | To generate the NEO fragments with artificial intron |

| | | |
|---|---|---|
| neo-intron346-P3 | gtattagaagaatatcctgatCTATTGATTAGCTTAGACTAGTAAAATAGC ACCTACtcaggtgaaaatattgttga | To generate the NEO fragments with artificial intron |
| Neo-TYCZ-P5 | catcaacaatattttcacctgaatcaggatattcttctaatacctggaatgctgttttcccggggat | To generate the NEO fragments with artificial intron |
| Neo-TYCZ-P3 | atgggtataaatgggctcgcgataatgtcgggcaatcaggtgcgacaatctatcgattg | To generate the NEO fragments with artificial intron |
| **Intermediate plasmids for crRNA constructs** | | |
| crMEL1-NT1-P5 | gat CAGATTGGGGAGTAGATTACTTAAA | the oligonucleotide pairs designed for spacer of each crRNA |
| crMEL1-NT1-P3 | gcc TTTAAGTAATCTACTCCCCAATCTG | |
| crMEL1-NT2-P5 | gat CAATAATGATTGGGTAGTGGCTGTT | the oligonucleotide pairs designed for spacer of each crRNA |
| crMEL1-NT2-P3 | gcc AACAGCCACTACCCAATCATTATTG | |
| crMEL1-T3-P5 | gat GTCCCAAACCATTATAGCTACCATG | the oligonucleotide pairs designed for spacer of each crRNA |
| crMEL1-T3-P3 | gcc CATGGTAGCTATAATGGTTTGGGAC | |
| crMEL1-T4-P5 | gat CTTCCAATCTACCAGTGGTTGCATT | the oligonucleotide pairs designed for spacer of each crRNA |
| crMEL1-T4-P3 | gcc AATGCAACCACTGGTAGATTGGAAG | |
| crMEL1-T5-P5 | gat AGTAATCTACTCCCCAATCTGCAAA | the oligonucleotide pairs designed for spacer of each crRNA |
| crMEL1-T5-P3 | gcc TTTGCAGATTGGGGAGTAGATTACT | |
| crMEL1-Control-P5 | gat AACAGCGCCTTAAAAGAACTAGAAA | the oligonucleotide pairs designed for spacer of each crRNA |
| crMEL1-Control-P3 | gcc TTTCTAGTTCTTTTAAGGCGCTGTT | |
| crNEO346-P5 | GAT acctgaatcaggatattcttctaat | the oligonucleotide pairs designed for spacer of each crRNA |
| crNEO346-P3 | GCC attagaagaatatcctgattcaggt | |
| crNEO299-P5 | GAT ccggggatcgcagtggtgagtaacc | the oligonucleotide pairs designed for spacer of each crRNA |
| crNEO299-P3 | GCC ggttactcaccactgcgatccccgg | |
| crNEO203-P5 | GAT gtctgaccatctcatctgtaacatc | the oligonucleotide pairs designed for spacer of each crRNA |
| crNEO203-P3 | GCC gatgttacagatgagatggtcagac | |

| | | |
|---|---|---|
| crNEO154-1-P5 | GAT ccatgtttcagaaacaactctggcg | the oligonucleotide pairs designed for spacer of each crRNA |
| crNEO154-1-P3 | GCC cgccagagttgtttctgaaacatgg | |
| crNEO154-2-P5 | GAT agaaacaactctggcgcatcgggct | the oligonucleotide pairs designed for spacer of each crRNA |
| crNEO154-2-P3 | GCC agcccgatgcgccagagttgtttct | |
| crNEO-Control-P5 | gat AACAGCGCCTTAAAAGAACTAGAAA | the oligonucleotide pairs designed for spacer of each crRNA |
| crNEO-Control-P3 | gcc TTTCTAGTTCTTTTAAGGCGCTGTT | |
| Tf1-835-gRNA-P5 | aac Atccaactaggtttaccattcttcttaa | the oligonucleotide pairs designed for spacer of each crRNA |
| Tf1-835-gRNA-P3 | gcc ttaagaagaatggtaaacctagttggat | |
| Tf1-1165-gRNA-P5 | aac Tgtcgttctcctttaaaaacttattgtt | the oligonucleotide pairs designed for spacer of each crRNA |
| Tf1-1165-gRNA-P3 | gcc aacaataagtttttaaaggagaacgaca | |
| Tf1-Control-gRNA-P5 | acc CAGACTATGCGTCGACAAGCCAGGCATT | the oligonucleotide pairs designed for spacer of each crRNA |
| Tf1-Control-gRNA-P3 | gcc AATGCCTGGCTTGTCGACGCATAGTCTG | |

**Construction of crRNA vectors to direct targeting by FnCpf1, LbCpf1, and LshCas13a**

| | | |
|---|---|---|
| pDUAL-PspXI-T7 | CCTCCAATCTTGTGTTCTTCAAA TAATACGACTCACTATAGG | Amplification of crRNA array cassettes inserted into linearized plasmids pDUAL-HFF1 |
| pDUAL-SpeI-T3 | CGCTAGGGATAACAGGGTAATAT AATTAACCCTCACTAAAGG | Amplification of crRNA array cassettes inserted into linearized plasmids pDUAL-HFF1 |
| pHL414-NheI-T7 | TGCAGCCCGGGGGATCCCAGCTGTAATACGACTCACTATAG | Amplification of crRNA array cassettes inserted into linearized plasmids of Tf1 retrotransposition reporters |
| pHL414-NheI-T3 | ATCGCCAGTCACTATGGCGTGCTtctagaAATTAACCCTCACTAAAG | Amplification of crRNA array cassettes inserted into linearized plasmids of Tf1 retrotransposition reporters |

| | | |
|---|---|---|
| pDUAL-SpeI-SK primer | ATCGACGATAAAAGAATCATCTTtctagaactagtggatc | Amplification of crRNA array cassettes inserted into linearized plasmid pDUAL-HFF1-FnCpf1 |
| pDUAL-SpeI-T3 | CGCTAGGGATAACAGGGTAATATAATTAACCCTCACTAAAGG | Amplification of crRNA array cassettesinserted into linearized plasmid pDUAL-HFF1-FnCpf1 |
| NheI-TYB-P5 | GGGGGATCCCAGCTGGCTAGCAATTAACCCTCACTAAAGG | Amplification of crRNA array cassettes inserted into linearized plasmid pHL414 |
| NheI-TYB-P3 | TCACTATGGCGTGCTGCTAGCTAATACGACTCACTATAGG | Amplification of crRNA array cassettes inserted into linearized plasmid pHL414 |
| **MEL1-repair fragment** | | |
| mel1-left-P5-new | AGGTTAAAACTCCAGAACATTAC | Amplification of repair fragment for *MEL1* deletion |
| mel1-left-P3-new | TCATGTGCTAGGTCGATTCTGG GAACAAGAAGTCAATATCGCTTAC | Amplification of repair fragment for *MEL1* deletion |
| mel1-right-P5-new | GTAAGCGATATTGACTTCTTGTTC CCAGAATCGACCTAGCACATGA | Amplification of repair fragment for *MEL1* deletion |
| mel1-right-P3-new | GCTATAGTAGAACGAGCCCCGA | Amplification of repair fragment for *MEL1* deletion |
| MEL1-P5 | AGGTTAAAACTCCAGAACATTAC | Confirmation *MEL1* deletion |
| MEL1-P3 | GCTATAGTAGAACGAGCCCCGA | Confirmation *MEL1* deletion |

**Supplementary Data S2**

## Interference with Tf1 retrotransposition by CRISPR-Cas12a leaving marginal transposition activities

| crRNA-plasmid | # of colonies | YE+Ura.+5-FOA --$10^5$ cell / mL | YE+Ura.+5-FOA+G418 --$10^6$ cell / mL | Transposition frequency(%) | Normalized - Transposition frequency(%) |
|---|---|---|---|---|---|
| | crNEO346-① | 1500 | 12 | 0.08 | |
| cr*NEO* 346 | crNEO346-② | 2500 | 14 | 0.06 | |
| | crNEO346-③ | 2795 | 19 | 0.07 | |
| | mean (crNEO346) | | | 0.07 | 0.1 |
| | crNEO-control-1-① | 2000 | 74 | 0.37 | |
| | crNEO-control-1-② | 2000 | 86 | 0.43 | |
| | crNEO-control-1-③ | 2000 | 160 | 0.80 | |
| cr*NEO* control | crNEO-control-2-① | 2600 | 259 | 1.00 | |
| | crNEO-control-2-② | 2200 | 228 | 1.04 | |
| | crNEO-control-2-③ | 2300 | 210 | 0.91 | |
| | mean (crNEO-control) | | | 0.76 | 1.0 |

## Prolonged crRNA targeting eliminating residual Tf1 retrotransposition by CRISPR-Cas12a

| crRNA-strain | # of colonies | YE+Ura.+5-FOA --$10^5$ cell / mL | YE+Ura.+5-FOA+G418 --$10^7$cell / mL | Transposition frequency(%) | Normalized - Transposition frequency(%) |
|---|---|---|---|---|---|
| | crNEO 346-① | 2200 | 1 | 0.000 | |
| cr*NEO* 346 | crNEO 346-② | 2000 | 1 | 0.001 | |
| | crNEO 346-③ | 2500 | 1 | 0.000 | |
| | mean (crNEO 346) | | | 0.000 | 0.00 |
| | crNEO control-① | 2500 | 1220 | 0.488 | |
| cr*NEO* control | crNEO control-② | 2500 | 1214 | 0.486 | |
| | crNEO control-③ | 2500 | 1257 | 0.503 | |
| | mean (crNEO control) | | | 0.492 | 1.00 |

**Interfering with Tf1 retrotransposition by CRISPR-Cas13a via targeting its RNA intermediates**

| crRNA-plasmid | # of colonies | YE+Ura.+5-FOA+G418 --$10^6$ cell / mL | YE+Ura.+5-FOA --$10^5$cell / mL | Transposition frequency(%) | Normalized -Transposition frequency(%) |
|---|---|---|---|---|---|
| Tf1-835 | Tf1-835-① | 820 | 2100 | 3.905 | |
| | Tf1-835-② | 800 | 2000 | 4.000 | |
| | Tf1-835-③ | 870 | 2000 | 4.350 | |
| | mean (Tf1-835-1) | | | 4.085 | 0.84 |
| Tf1-1165 | Tf1-1165-① | 340 | 1700 | 2.000 | |
| | Tf1-1165-② | 400 | 2150 | 1.860 | |
| | Tf1-1165-③ | 400 | 2020 | 1.980 | |
| | mean(Tf1-1165-3) | | | 1.947 | 0.40 |
| Tf1-control | Tf1-control-① | 970 | 1900 | 5.105 | |
| | Tf1-control-② | 1020 | 2000 | 5.100 | |
| | Tf1-control-③ | 910 | 2100 | 4.333 | |
| | mean (Tf1-control) | | | 4.846 | 1.00 |
